# *Rickettsia typhi* Possesses Phospholipase A_2_ Enzymes that Are Involved in Infection of Host Cells

**DOI:** 10.1371/journal.ppat.1003399

**Published:** 2013-06-20

**Authors:** M. Sayeedur Rahman, Joseph J. Gillespie, Simran Jeet Kaur, Khandra T. Sears, Shane M. Ceraul, Magda Beier-Sexton, Abdu F. Azad

**Affiliations:** 1 Department of Microbiology and Immunology, University of Maryland School of Medicine, Baltimore, Maryland, United States of America; 2 Virginia Bioinformatics Institute at Virginia Tech, Blacksburg, Virginia, United States of America; Tufts University School of Medicine, United States of America

## Abstract

The long-standing proposal that phospholipase A_2_ (PLA_2_) enzymes are involved in rickettsial infection of host cells has been given support by the recent characterization of a patatin phospholipase (Pat2) with PLA_2_ activity from the pathogens *Rickettsia prowazekii* and *R. typhi*. However, *pat2* is not encoded in all *Rickettsia* genomes; yet another uncharacterized patatin (Pat1) is indeed ubiquitous. Here, evolutionary analysis of both patatins across 46 *Rickettsia* genomes revealed 1) *pat1* and *pat2* loci are syntenic across all genomes, 2) both Pat1 and Pat2 do not contain predicted Sec-dependent signal sequences, 3) *pat2* has been pseudogenized multiple times in rickettsial evolution, and 4) ubiquitous *pat1* forms two divergent groups (*pat1A* and *pat1B*) with strong evidence for recombination between *pat1B* and plasmid-encoded homologs. In light of these findings, we extended the characterization of *R. typhi* Pat1 and Pat2 proteins and determined their role in the infection process. As previously demonstrated for Pat2, we determined that 1) Pat1 is expressed and secreted into the host cytoplasm during *R. typhi* infection, 2) expression of recombinant Pat1 is cytotoxic to yeast cells, 3) recombinant Pat1 possesses PLA_2_ activity that requires a host cofactor, and 4) both Pat1 cytotoxicity and PLA_2_ activity were reduced by PLA_2_ inhibitors and abolished by site-directed mutagenesis of catalytic Ser/Asp residues. To ascertain the role of Pat1 and Pat2 in *R. typhi* infection, antibodies to both proteins were used to pretreat rickettsiae. Subsequent invasion and plaque assays both indicated a significant decrease in *R. typhi* infection compared to that by pre-immune IgG. Furthermore, antibody-pretreatment of *R. typhi* blocked/delayed phagosomal escapes. Together, these data suggest both enzymes are involved early in the infection process. Collectively, our study suggests that *R. typhi* utilizes two evolutionary divergent patatin phospholipases to support its intracellular life cycle, a mechanism distinguishing it from other rickettsial species.

## Author Summary

Typhus Group (TG) rickettsiae include *Rickettsia typhi* and *R. prowazekii*, the etiological agents of murine and epidemic typhus, respectively. The rickettsial obligate intracellular life cycle complicates conventional approaches to genetic manipulation, impeding characterization of molecules underlying pathogenesis. Phospholipase A_2_ (PLA_2_) enzymes are factors that likely mediate such processes as host entry, phagosomal escape, and host lysis for cell-to-cell spread. Here, we demonstrate across 46 *Rickettsia* genomes that genes encoding two PLA_2_ enzymes (*pat1* and *pat2*) have divergent evolutionary histories and discordant selective constraints, with *pat2* deleted from most other non-TG rickettsiae genomes. As previously determined for Pat2, we show here that *R. typhi* Pat1 is secreted into the host cell cytoplasm during infection and requires a host cofactor for enzymatic activity. Like Pat2, recombinant Pat1 protein is cytotoxic to yeast cells. Pretreatment of *R. typhi* with anti-Pat1 or anti-Pat2 antibody results in a significant decrease in rickettsial infection, implicating roles for both Pat1 and Pat2 during the early stage of *R. typhi* host cell infection. Collectively, our work suggests that *R. typhi* utilizes two evolutionary divergent phospholipases during its intracellular life cycle, a mechanism distinguishing TG rickettsiae-associated cell biology and pathogenesis from other rickettsioses, particularly those associated with Spotted Fever Group pathogens.

## Introduction

Bacterial species of the genus *Rickettsia* (*Alphaproteobacteria*: Rickettsiales: Rickettsiaceae) are Gram-negative, obligate intracellular bacteria with a life cycle typically involving arthropod vectors and vertebrate hosts [1]. Some members of the genus *Rickettsia* are serious human pathogens, such as the agents of epidemic typhus (*R. prowazekii*) and Rocky Mountain Spotted Fever (*R. rickettsii*) [1]. Included with *R. prowazekii* in the typhus group (TG) rickettsiae, *R. typhi* is the causative agent of murine typhus and is transmitted by fleas throughout the world [2]. Murine typhus presents as a mild to severe flu-like illness, with over 70% of patients requiring hospitalization, and if left untreated, can be fatal in humans [3], [4], [5]. Murine typhus is endemic in the continental US and is substantially re-emerging in southern Texas and California, where the current level of reported human cases is continuing to occur with high prevalence [2], [6], [7], [8], [9].

The obligate intracellular life cycle of *Rickettsia* spp. involves entry into host cells by phagocytosis (or induced phagocytosis for non-phagocytic cell types), rapid escape from the phagocytic vacuole into the host cytoplasm to evade phagosome-lysosome fusion, replication within the host cytoplasm, and exit from the host cell by actin-mediated motility (e.g., Spotted Fever Group rickettsiae) or lysis of host cells (e.g., TG rickettsiae) [1], [10]. The genomes of nearly 50 rickettsial species have been sequenced and provide many insights into their biology [11]. However, very little is known regarding the molecular mechanisms of rickettsial intracellular growth and pathogenesis due to limited tools and approaches for genetic manipulation [12], [13].

Rickettsial phospholipase A_2_ (PLA_2_) activity has long been proposed to mediate rickettsial entry into host cells, escape from the phagosome and lysis of host cells [14], [15], [16], [17], [18]. However, the corresponding rickettsial gene(s) encoding PLA_2_ and the exact mechanism of such enzymes (e.g., host/vector range, substrate specificity, and activity period during life cycle) in rickettsial intracellular life is not well understood. Recently, we reported that the *R. typhi* genome possesses two genes encoding patatin (Pat)-like PLA_2_ proteins: RT0590 (Pat1) and RT0522 (Pat2), and demonstrated that Pat2 possesses PLA_2_ activity [19]. A subsequent report demonstrated that the Pat2 homolog of *R. prowazekii* (RP534) also possesses PLA_2_ activity [20]. While Pat1 homologs are encoded in all sequenced *Rickettsia* genomes, Pat2 is known from a much narrower range of species [19], [21]. Genes conserved in TG rickettsiae, relative to the larger rickettsial genomes, likely underlay important factors distinguishing TG rickettsiae cell biology and pathogenesis from other rickettsioses [22]. Thus, the potential utilization of two divergent Pat enzymes throughout the bacterial infection cycle may indicate a mechanism unique to TG rickettsiae.

Elucidating the role of patatins in rickettsial infection is important for determining the factors underlying rickettsial obligate intracellular infection and pathogenesis. In our previous report [19] we showed that the cytotoxicity and PLA_2_ activity of *R. typhi* Pat2 is relatively low compared to that of *Pseudomonas aeruginosa* ExoU, a secreted cytotoxin and virulence factor with PLA_2_ activity [23], [24]. This suggests that *R. typhi* Pat2 may be necessary to support intracellular survival without affecting host cell integrity, at least until the rickettsiae are ready to lyse the host cell and spread to cause further infection. In this communication, we report the further characterization of PLA_2_ activity for both Pat1 and Pat2 of *R. typhi*, and also provide important properties of both enzymes during *R. typhi* infection of host cells. Using bioinformatics and comparative genomics, important evolutionary characteristics of both patatins are illustrated and suggest differential strategies for patatin utilization across species of *Rickettsia*. Collectively, these new data shed light on the molecular dynamics orchestrating the *R. typhi* obligate intracellular life cycle within eukaryotic cells.

## Results

### Conservation of *Rickettsia* patatins

**Figure 1:**
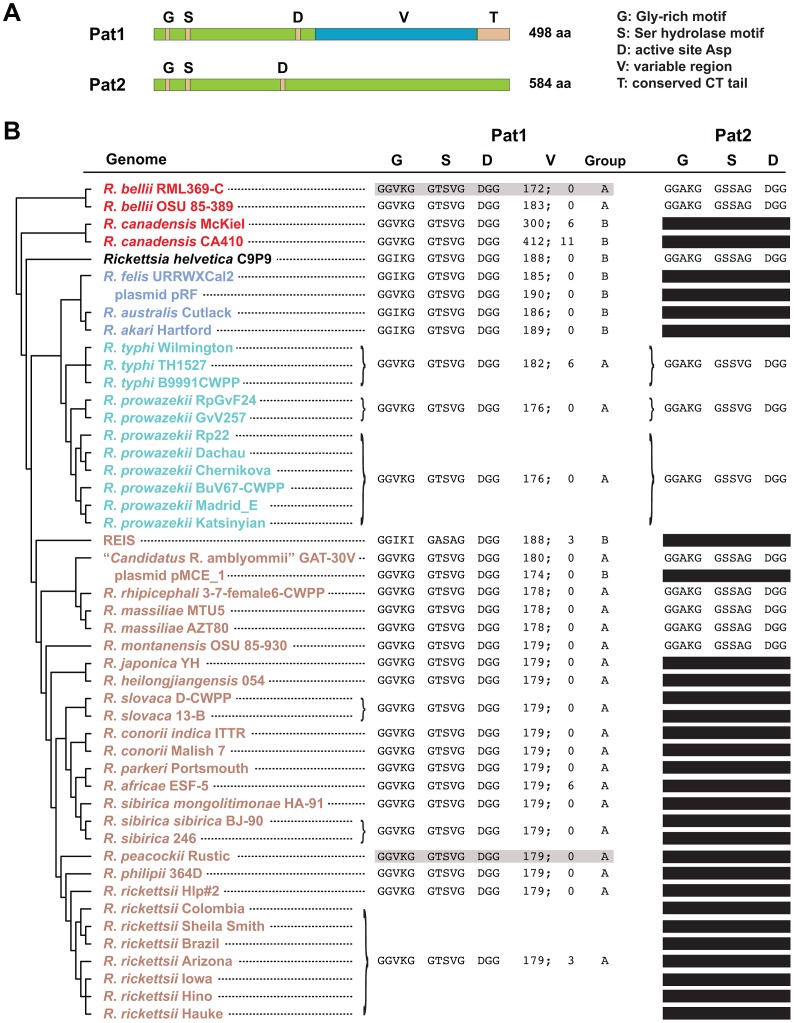
*In silico* characteristics of rickettsial patatins (Pat1 and Pat2). **A**) Schema depicting the major features of *R. typhi* Pat1 (RT0590, YP_067537) and Pat2 (RT0522, YP_067473) proteins. The conserved Glycine-rich motif (G), Serine hydrolase motif (S) and active site Aspartate (D) of patatins are depicted for both proteins, with a variable region (V) and conserved C-terminal tail (T) illustrated for Pat1. Neither protein was predicted to contain an N-terminal Sec-dependent secretion signal (see text). **B**) Comparative analysis of Pat1 and Pat2 proteins across 46 *Rickettsia* genomes. Phylogeny at left is based on whole genome analysis [73], with rickettsial groups as follows: red, ancestral group; aquamarine, typhus group; blue, transitional group, brown, spotted fever group [26], [74]. *R. helvetica*, previously categorized as spotted fever group rickettsiae, is shown as *incertae sedis*. Plasmids pRF (*R. felis*) and pMCE_1 (“*Candidatus R. amblyommii*”), which encode Pat1 homologs, are also listed (but not included in the phylogeny estimation). The sequences within the conserved G, S and D regions for both Pat1 and Pat2 are shown. For Pat1, the length of the V region is provided, followed by number of predicted repeats [66]. The group designation (A or B) for each Pat1 sequence is provided based on phylogeny estimation (see **Fig. 2A**). For Pat1, sequences highlighted gray (*R. bellii* str. RML369-C and *R. peacockii* str. Rustic) depict the proteins comprised of two ORFs split near the C-terminus (see **Fig. 2B**). For Pat2, black bars depict pseudogenes, which comprise short annotated ORFs with strong homology to full length Pat2 proteins or smaller fragments identified using TBLASTN. Accession numbers for all sequences are provided in **Table S1**.

Informatics analysis identified two Pat-like phospholipases encoded within *Rickettsia* genomes, both of which contain the features typical of Pat proteins (**Fig. 1A**). The rickettsial Pats (Pat1 and Pat2) are larger than the average size of bacterial Pats, with the C-terminal halves of both proteins highly divergent and dissimilar to the C-terminal regions of other prokaryotic and eukaryotic Pats (data not shown). Comparative analysis of 46 *Rickettsia* genomes indicates that Pat1 is a ubiquitous protein that is likely essential for rickettsial biology (**Fig. 1B**). Alternatively, Pat2 is encoded in only 24% of *Rickettsia* genomes, with apparent loss of the *pat2* gene occurring multiple times throughout rickettsial evolution. Pseudogenization of *pat2* is an active process, as indicated in some genomes by short annotated ORFs with strong homology to portions of full length Pat2 proteins, or by even smaller fragments identified using TBLASTN (data not shown).

*Rickettsia* Pat1 and Pat2 are very divergent from one another. The best scoring matches to the NCBI Conserved Domains Database (CDD) for Pat1 and Pat2 are cd07199 and cd07207, respectively. cd07199 includes PNPLA8, PNPLA9, and Pat17 patatin-like phospholipases, while cd07207 is typified by the secreted bacterial proteins ExoU (*Pseudomonas aeruginosa*) and VipD (*Legionella pneumophila*). Within-genome BLASTP searches (e.g., using Pat1 as a query to detect Pat2, and vice versa) do not result in significant matches, and inspection of the top 500 hits in BLASTP searches against the NCBI database of non-redundant (nr) protein sequences (using either Pat1 or Pat 2 sequences as queries) suggest that distant homologs of both proteins do not overlap. This sequence divergence between Pat1 and Pat2, coupled with their distribution patterns across *Rickettsia* genomes, suggests different functions for Pat1 and Pat2 in rickettsial biology.

### *Rickettsia pat1* and *pat2* have different evolutionary patterns

The top scoring BLASTP hits against the nr database (excluding *Rickettsia*) for *R. typhi* Pat1 (the lycophyte *Selaginella moellendorffii*, 122 bits, 35% aa identify) and Pat2 (*L. pneumophila* str. Corby, 169 bits, 39% aa identity) illustrate that both proteins are highly divergent from their closest homologs in other organisms. Phylogeny estimation of Pat1-like sequences placed the rickettsial proteins in a clade with Pats from other intracellular bacterial species, including “*Candidatus* Odyssella thessalonicensis” (Rickettsiales), *Coxiella burnetii* (*Gammaproteobacteria*), “*Candidatus* Amoebophilus asiaticus” (Bacteroidetes), and Rickettsiales endosymbiont of *Trichoplax adhaerens* (**Fig. S1**). Phylogeny estimation of Pat2-like sequences placed the rickettsial proteins in a clade with ExoU (*Pseudomonas* spp.), VipD (*Legionella* spp.) and related Pats from other *Gammaproteobacteria* and *Betaproteobacteria* species (**Fig. S2**). Thus, neither Pat1 nor Pat2 of *Rickettsia* spp. show a pattern of vertical descent in the bacterial species tree, with Pats and other phospholipases from other *Alphaproteobacteria* (including Anaplasmataceae, the sister family to *Rickettsia* spp.) being unrelated.

The chromosomal locations of both *pat1* and *pat2* (full length or pseudogene) are conserved across all *Rickettsia* genomes, suggesting both genes were present in the last common ancestor of the analyzed genomes (data not shown). Despite rampant *pat2* pseudogenization across many *Rickettsia* genomes, the 11 full-length Pat2 proteins are conserved in sequence (81% avg. aa identity) with negligible length variation. Phylogeny estimation of the *Rickettsia* Pat2 sequences is congruous with the *Rickettsia* species tree (**Fig. S2**), suggesting independent gene loss and a lack of recombination at this conserved locus. The absence of *pat2* genes in the pool of rickettsial mobile genetic elements (*Rickettsia* mobilome) supports these observations.

**Figure 2:**
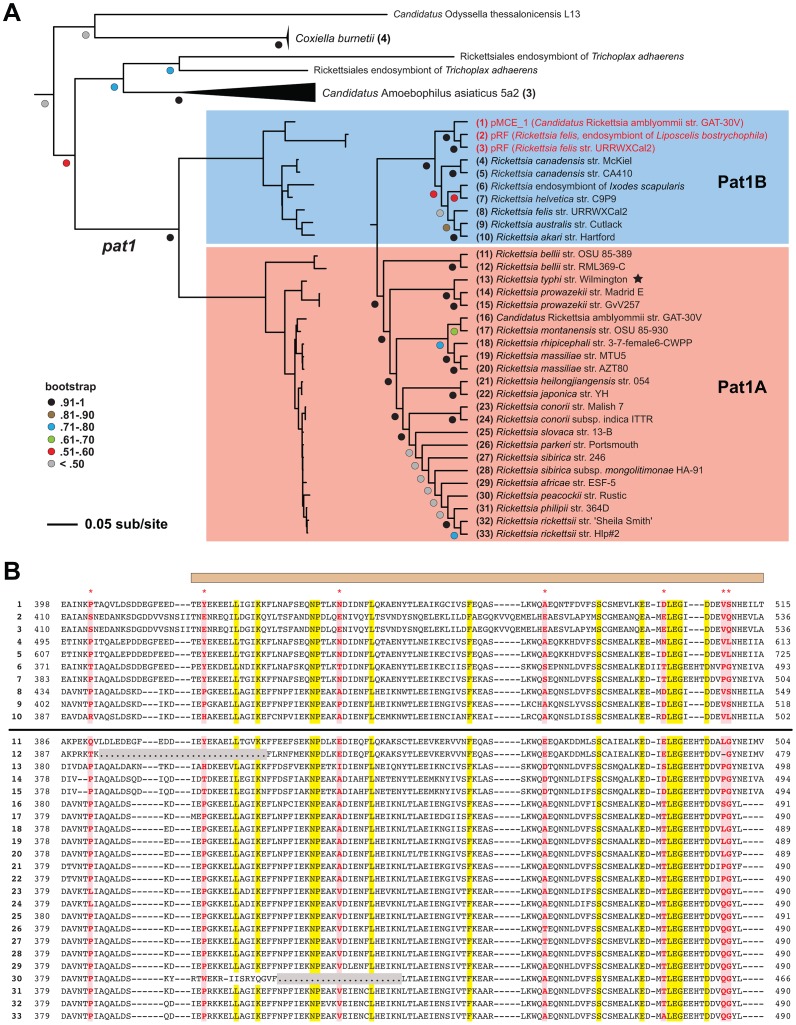
Evolutionary analysis of rickettsial Pat1 proteins. **A**) Phylogeny estimation of rickettsial Pat1 proteins, with closest non-*Rickettsia* clades shown. The complete tree containing 93 ExoU-like patatins is shown in **Fig S2**. See Materials and Methods for details of phylogenetic analysis. The *Rickettsia* Pat1 sequences (*n* = 33) form two well-supported clades (Pat1A and Pat1B). The Pat1B group (1–10, shaded blue) contains the plasmid-encoded Pat1 proteins (colored red), Pat1 of the *R. canadensis* strains, and the proteins from REIS, *R. helvetica* (*incertae sedis*) and the transitional group rickettsiae. The Pat1A group (11–33, shaded blue) contains *R. typhi* RT0590 (noted with a black star), the *R. prowazekii* and *R. bellii* proteins, and Pat1 from the majority of the spotted fever group rickettsiae. **B**) Comparative analysis of the C-terminal tails for 33 rickettsial Pat1 proteins. See text for alignment details. The sequences are listed (1–33) as they appear in the phylogenetic tree in panel **A**. The horizontal bar distinguishes Pat1B (upper) from Pat1A (lower) sequences. The tan bar above the alignment illustrates the delineation between the tail region and the variable region of Pat1 sequences. Coordinates for each sequence are provided at left and right. Conserved positions across the alignment are highlighted yellow, with positions predicted to evolve under positive selection [71] highlighted red and noted at the top of the alignment by asterisks. Positions depicted with dots and highlighted gray illustrate missing sequence for Pat1 proteins encoded by two ORFs (*R. bellii* str. RML369-C and *R. peacockii* str. Rustic).

Alternatively, despite being encoded in all *Rickettsia* genomes, Pat1 sequences are extraordinarily divergent in sequence length and composition, particularly in the variable (V) region that comprises the majority of the C-terminal half of the protein. The largest Pat1 sequences (*R. canadensis* genomes) contain from six to eleven Glu-rich repeats (EENIENNQETINKPITQALEPDDEDFEE) within the V region, with smaller cryptic repeats predicted in other Pat1 sequences (data not shown). Phylogeny estimation of Pat1 proteins yielded a tree that is highly incongruent with the *Rickettsia* species tree (shown in **Fig. 1**), a result previously shown in an analysis of only nine *Rickettsia* genomes [25]. Our robust phylogeny estimation placed the Pat1 sequences from 46 *Rickettsia* genomes into two well-supported groups (Pat1A and Pat1B) (**Fig. 2A**). The Pat1B sequences include plasmid-encoded Pat1 proteins (pRF of *R. felis* and pMCE_1 of “*Candidatus* R. amblyommii”), the larger *R. canadensis* proteins, and the proteins from REIS, *R. helvetica* (*incertae sedis*) and the transitional group rickettsiae [26]. The remaining Pat1 proteins form the Pat1A group, which superficially corroborates the *Rickettsia* species tree (minus the sequences from Pat1B). This phylogeny estimation, coupled with the conserved chromosomal locale of all *pat1* genes, strongly implicates multiple recombination events between rickettsial plasmid-encoded *pat1* and the chromosomal-encoded *pat1* genes within the Pat1B group.

Despite being highly divergent from one another, Pat1A and Pat1B proteins contain a conserved C-terminal tail (T) containing approximately 100 aa residues (**Fig. 2B**). This further fortifies the role of recombination in shaping Pat1 diversity across *Rickettsia* genomes. Six residues within the T region, plus an additional nearby residue in the V region, were predicted to have evolved under positive selection. This implicates a possible function for the T region, perhaps in contact with host cell molecules, or in mediating the secretion of Pat1 out of the rickettsial cell (see **DISCUSSION**).

### Expression and translocation of *R. typhi* Pat1 protein

**Figure 3:**
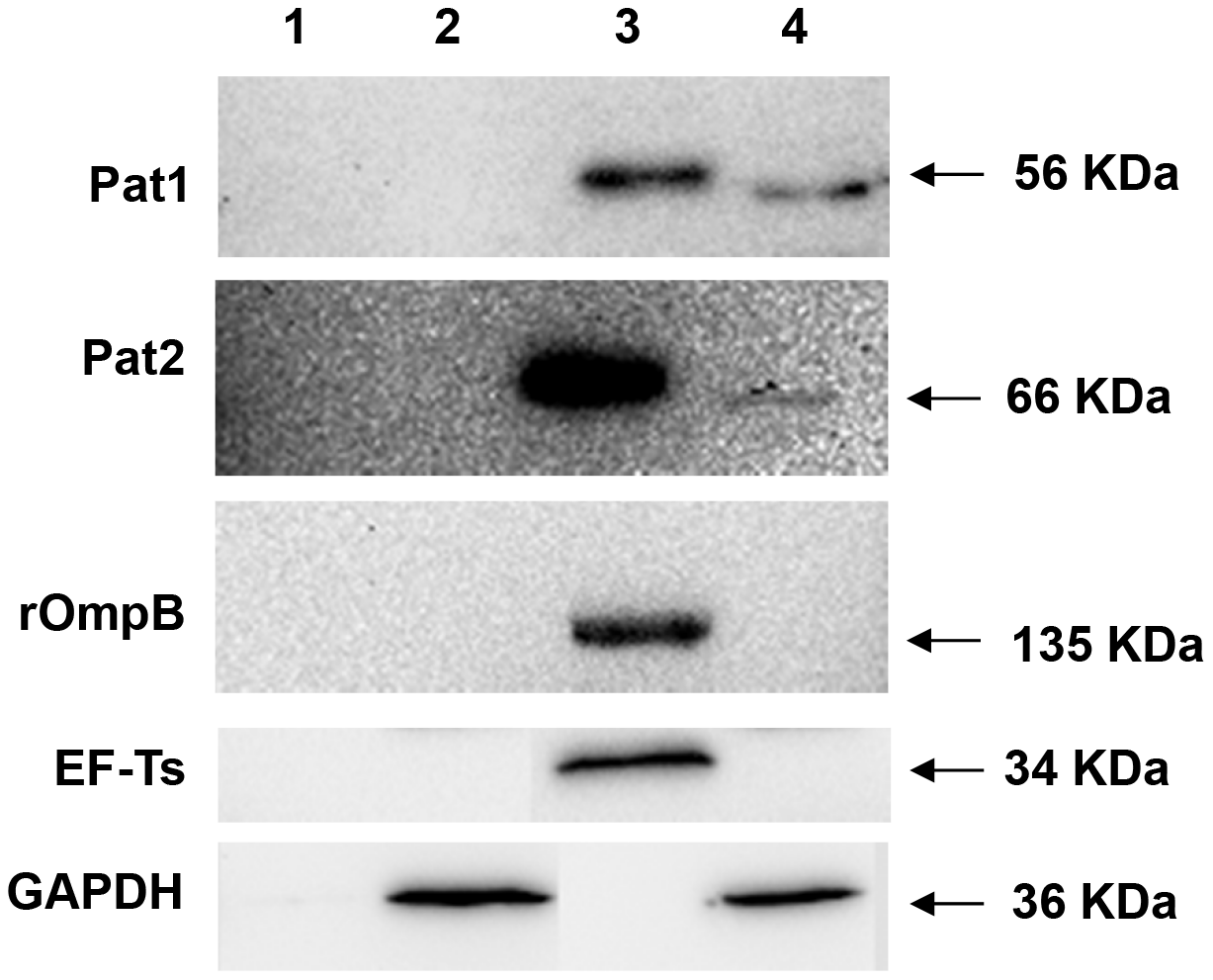
Translocation of Pat1 during *R. typhi* infection of Vero76 cells by Western blotting. *R. typhi* infected or uninfected Vero76 grown for 48 hr were fractionated by 0.1% Triton X-100 treatment into pellet containing intact rickettsia with host cell debris and supernatant containing rickettsial secreted protein with host soluble proteins and was probed with rabbit anti-Pat1 antibody, rabbit anti-Pat2 antibody (as positive control for *R. typhi* secreted protein), rabbit anti-rOmpB antibody (as control for *R. typhi* surface protein), rabbit anti-EF-Ts antibody (as control for *R. typhi* cytoplasmic protein) or mouse anti-GAPDH monoclonal antibody (as control for host cytoplasmic protein). **Lane1**: pellet of uninfected Vero76; **Lane2**: supernatant of uninfected Vero76; **Lane3**: pellet of *R. typhi* infected Vero76; **Lane4**: supernatant of *R. typhi* infected Vero76. The size of the expected protein bands is shown on the right.

To determine the expression of Pat1 protein and its secretion into host cells during *R. typhi* infection, we prepared two cellular fractions, pellet and supernatant, from uninfected or *R. typhi*-infected Vero76 cells after incubation for 48 h. The pellet contained intact rickettsiae along with host cell debris. The supernatant contained the rickettsial secreted proteins, as well as the host soluble proteins. Both fractions from uninfected or *R. typhi*-infected Vero76 cells were probed with antibodies raised against Pat1, Pat2, rOmpB (Sca5), EF-Ts or GAPDH. GAPDH, which is a host cytoplasmic protein, was detected in the supernatant of both the uninfected and infected cells (**Fig. 3**, Lanes 2 and 4). The control EF-Ts, a soluble rickettsial cytoplasmic protein, was found only in the pellet of infected cells (**Fig. 3**, Lane 3), supporting that EF-Ts remained associated with *R. typhi* and that *R. typhi* remained intact during fractionation. We observed that rOmpB, which is a rickettsial outer membrane protein [27], was only present in the pellet of infected cells (**Fig. 3**, Lane3). This suggests rOmpB was not stripped and solubilized by 0.1% Triton X-100 treatment, but remained associated with the *R. typhi* cell surface. However, the positive control for *R. typhi* secreted protein Pat2 [19] and Pat1 were present in both the pellet (**Fig. 3**, Lane 3) and supernatant (**Fig. 3**, Lane 4) of infected cells. We also observed that both pellet-associated Pat1 and Pat2 (containing intact rickettsiae) migrated slightly slower than supernatant-associated proteins (containing rickettsial secreted proteins into host cytoplasm). These data suggest that Pat proteins are expressed and possibly processed during secretion into the host cell cytoplasm.

### Subcellular localization of *R. typhi* Pat1 and Pat2 proteins

**Figure 4:**
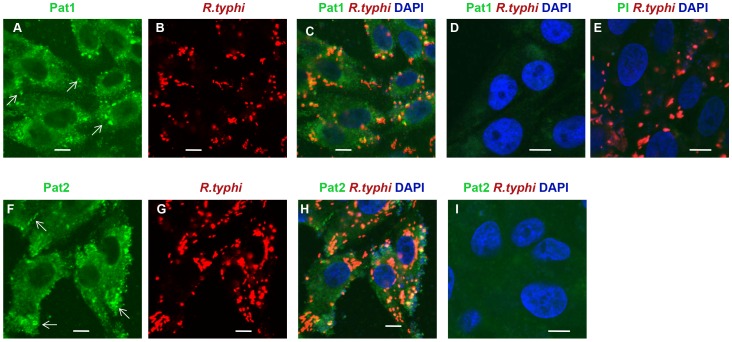
Secretion of Pat1 and Pat2 into host cells. Cells were fixed with 4% paraformaldehyde at 24 h postinfection and immunolabeled with anti-Pat1 (panels A to C for *R. typhi* infected and panel D for uninfected) or anti-Pat2 (panels F to H for *R. typhi* infected and panel I for uninfected) rabbit antibodies (at 1∶200 dilution) and anti-*R. typhi* rat serum (at 1∶500 dilution) as primary antibodies. *R. typhi* infected Vero76 cells were also labeled similarly with rabbit pre-immune serum (at 1∶200 dilution) and anti-*R. typhi* rat serum (at 1∶500 dilution) shown in panel E. The anti-rat-Alexa Fluor-594 (red) and anti-rabbit-Alexa Fluor-488 (green) antibodies were used as secondary antibodies. The cell nuclei were stained with DAPI (blue). Samples were viewed under a LSM5DUO confocal microscope and images were processed using ZEN imaging and analysis software. The white arrows showed punctate structure indicating translocation of Pat1 or Pat2 from rickettsiae into host cell cytoplasm. Scale Bar = 5 µm.

To further demonstrate the translocation of Pat1 (as shown in **Fig. 3**) and Pat2 [19], we analyzed the subcellular localization of both proteins during *R. typhi* infection in host cells using immunofluorescence assay (IFA). *R. typhi*-infected Vero76 cells were immunolabelled with antibodies against Pat1, Pat2 or *R. typhi*. While Pat1 and Pat2 co-localized with *R. typhi* (**Fig. 4A–C and F–H**), the formation of punctate structures throughout the host cell cytoplasm was also observed, indicating the translocation of both Pat1 and Pat2 from rickettsiae into host cytoplasm. For control, infected cells immunolabelled with preimmune serum (**Fig. 4E**), as well as uninfected Vero76 cells immunolabelled with anti-Pat antibodies (**Fig. 4D, I**), did not reveal a similar specific staining. Together, these data suggest that *R. typhi* Pat1 and Pat2 are translocated from rickettsiae into the host cell cytoplasm during infection.

### Cytotoxicity assay by yeast viability loss

**Figure 5:**
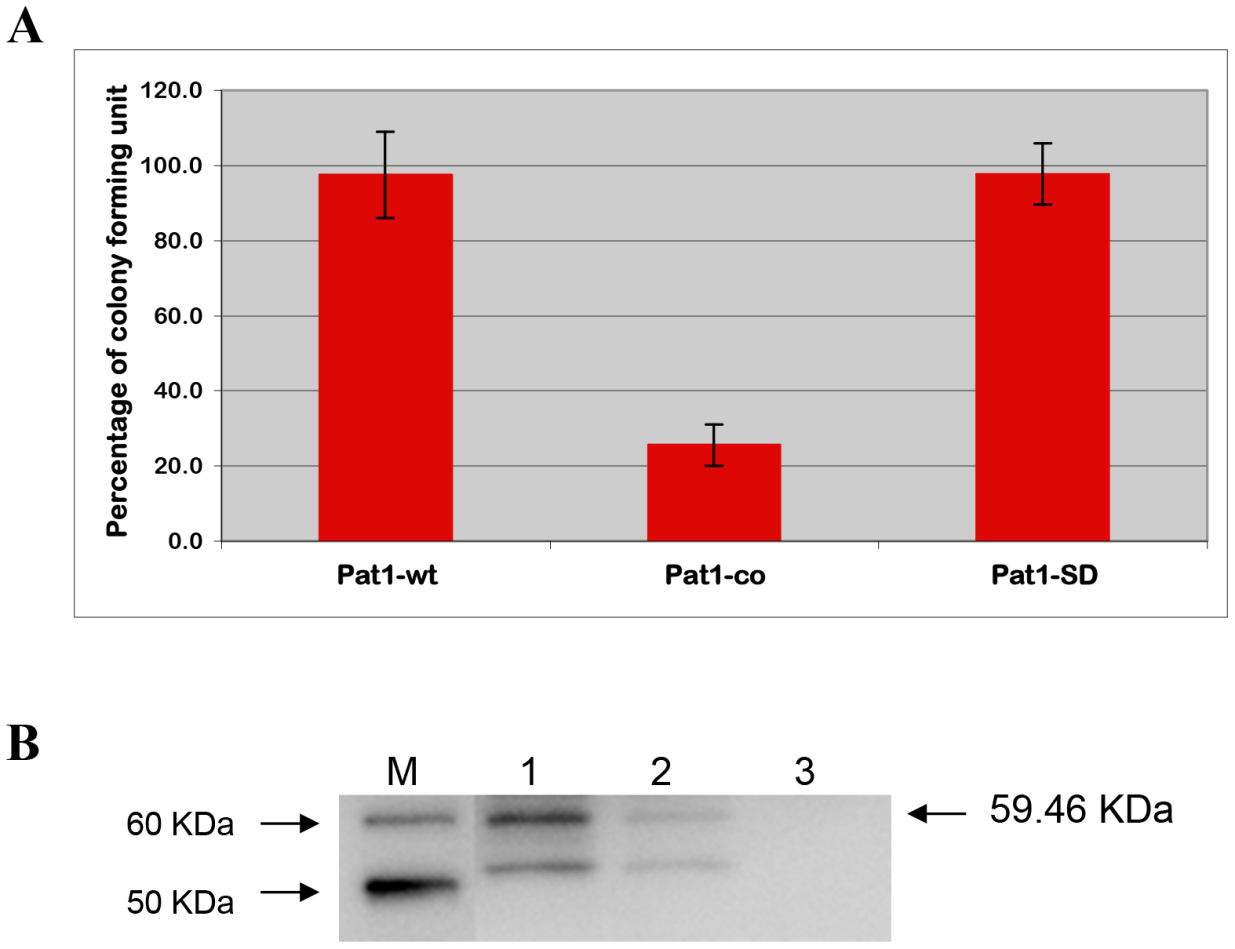
Cytotoxicity in yeast strain INVSc1 by CFU assay. The yeast strain INVSc1 transformed with the plasmids carrying genome sequence of *pat1* (Pat1-wt), carrying codon optimized sequence of *pat1* (Pat1-co) or carrying mutation at catalytic sites of *pat1-co* (Pat1-SD). **A**) The percentage of CFU in transformed yeast cells was determined as described (see Materials and Methods). **B**) Western blot analysis of the expression of Pat1 and its mutant derivatives in INVSc1 under inducing condition (SC-U+Gal medium). The total proteins from yeast cells carrying the appropriate plasmid were probed with anti-Pat1 antibody using WesternBreeze chemiluminescent immunodetection kit (Invitrogen). LaneM: MagicMark XP Western Protein Standard (Invitrogen), Lane1: Pat1-SD/INVSc1, Lane2: Pat1-co/INVSc1, Lane3: Pat1-wt/INVSc1. The size of the expected recombinant protein (including C-terminal V5 epitope and 6× His tag) is mentioned on the left (59.46 kDa). The bands below the expected protein size may have resulted from degradation of recombinant protein or nonspecific binding to proteins from yeast cell lysate.

Using a genetically tractable heterologous model system, the yeast *Saccharomyces cerevisiae* [23], [28], [29], [30], [31], [32], we previously demonstrated the cytotoxicity of *R. typhi* Pat2 [19]. To determine the potential cytotoxic effect of *R. typhi* Pat1, we again implemented the yeast model system. Yeast transformants with Pat1-wt (carrying the genome sequence of *pat1*) showed unaltered growth on both inducing (SC-U+Gal) and repressing (SC-U+Glu) agar, as determined by percentage CFU (**Fig. 5A**). We checked the expression of Pat1-wt in INVSc1 cells under inducing conditions and found no detectable expression of Pat1 protein in yeast cells (**Fig. 5B**, Lane 3). As *Rickettsia* spp. genomes are AT-rich (the *R. typhi* genome base composition is 28.9% GC) relative to *S. cerevisiae* (38.2% GC), we elected to codon optimize *pat1* for expression in eukaryotic cells [19]. The yeast strain INVSc1, transformed with plasmid Pat1-co (carrying the codon optimized sequence of *pat1*), expressed the expected protein (**Fig. 5B**, Lane 2), showing a substantial reduction in yeast growth (25.6±5.5%) on inducing agar with respect to repressing agar (**Fig. 5A**). These data indicate that the expression of Pat1 is cytotoxic to yeast cells.

The Ser/Asp catalytic dyad of patatin-like PLA_2_ enzymes is required for cytotoxicity [19], [23], [29], [30]. We implemented site-directed mutagenesis to examine the contribution of the Ser/Asp catalytic dyad in Pat1-mediated cytotoxicity. The plasmid Pat1-SD was constructed, with Ser-51 (serine hydrolase motif) and Asp-199 (active site aspartate) residues of *pat1-co* both replaced with alanine. The yeast strain INVSc1, transformed with plasmid Pat1-SD, expressed the expected proteins (**Fig. 5B**, Lane 1), with *S. cerevisiae* growth nearly completely restored (97.7±8.1%) as determined by the CFU assay (**Fig. 5A**). Collectively, these data indicate that the cytotoxicity caused by the expression of Pat1 in yeast is abolished by mutation at the predicted Ser/Asp catalytic sites of Pat1, supporting the inference that *R. typhi* Pat1 is a functional patatin.

### Demonstration of phospholipase A activity of Pat1

**Figure 6:**
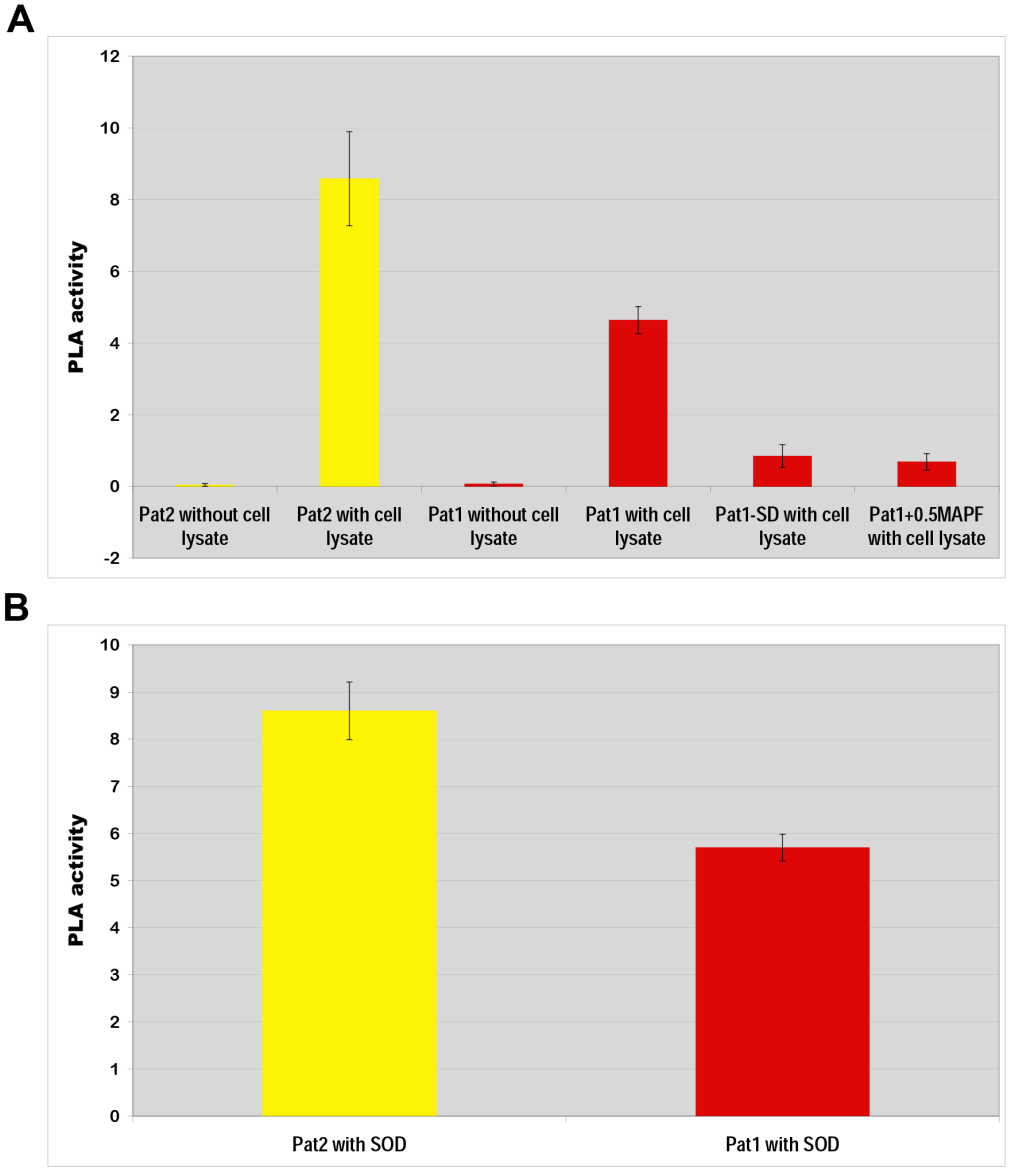
Phospholipase A activity assay of recombinant proteins. The phospholipase A (PLA) activity was determined via calculation of fluorescent emission/µg recombinant protein/min (see Materials and Methods). Error bars represent standard errors of the means. **A**) The PLA activity of recombinant proteins Pat2 or Pat1 increased significantly (P<0.05[two-tail t-test]) in presence of Vero76 cell lysate compared to that in absence of Vero76 cell lysate. The PLA activity of Pat1 was found to be significantly different (P<0.05[two-tail t-test]) from that of mutant Pat1-SD or in presence of 0.5 µM MAPF (PLA_2_ inhibitor). **B**) PLA activity assay of recombinant proteins: Pat2 and Pat1 in presence of bovine liver superoxide dismutase (SOD) as described in materials and methods.

To determine phospholipase A (PLA) activity of Pat1, we produced recombinant protein Pat1 in an *E. coli* protein expression system. We also used recombinant Pat2 as a positive control [19]. We observed that the recombinant protein (Pat1 or Pat2) exhibited elevated PLA activity in the presence of Vero76 cell lysate (**Fig. 6A**), indicating that Pat1 requires a eukaryotic activator for PLA activity. This is consistent with our previous results for Pat2 [19], although the PLA activity of Pat1 is slightly less than that of Pat2.

We further aimed to identify a possible eukaryotic factor required for the activation of *R. typhi* Pat1 and Pat2. The bovine liver superoxide dismutase (SOD) has previously been reported to function as an activator of *P. aeruginosa* ExoU [33]. Therefore, we evaluated the impact of SOD on Pat1 and Pat2 function. The results suggest that bovine liver SOD activates Pat1 and Pat2 PLA activity (**Fig. 6B**), with the level of activation similar to that observed by Vero76 cell lysate (**Fig. 6A**). Furthermore, the pattern of activation by SOD for both enzymes' PLA activity (Pat2>Pat1) is consistent with that observed by host cell lysate.

To examine the effect of the Ser/Asp catalytic residues in Pat1 mediated PLA activity, we produced the mutant derivative protein Pat1-SD (described above for the yeast model) in an *E. coli* protein expression system. The Pat1-SD recombinant protein exhibited significant (P<0.05 [two-tail t-test]) decrease of PLA activity (**Fig. 6A**). These data, which showed the effect of mutagenesis of catalytic Ser/Asp sites on PLA activity, clearly suggest Pat1 is a PLA enzyme.

To determine whether the inhibitors of calcium-independent (iPLA_2_) and calcium-dependent cytosolic (cPLA_2_) PLA_2_ activity block Pat1 function, we evaluated Pat1 PLA activity in the presence of methyl arachidonyl fluorophosphonate (MAPF), an irreversible inhibitor of both iPLA_2_ and cPLA_2_ [19],[30],[34],. At a concentration of 0.5 µM, MAPF significantly (P<0.05 [two-tail t-test]) reduced the PLA activity of Pat1 (**Fig. 6A**). These data further indicate that Pat1 possesses PLA activity, as this function can be blocked by iPLA_2_ and cPLA_2_ inhibitors.

### Elucidation of Pat1 and Pat2 function during the early stage of host cell infection

**Figure 7:**
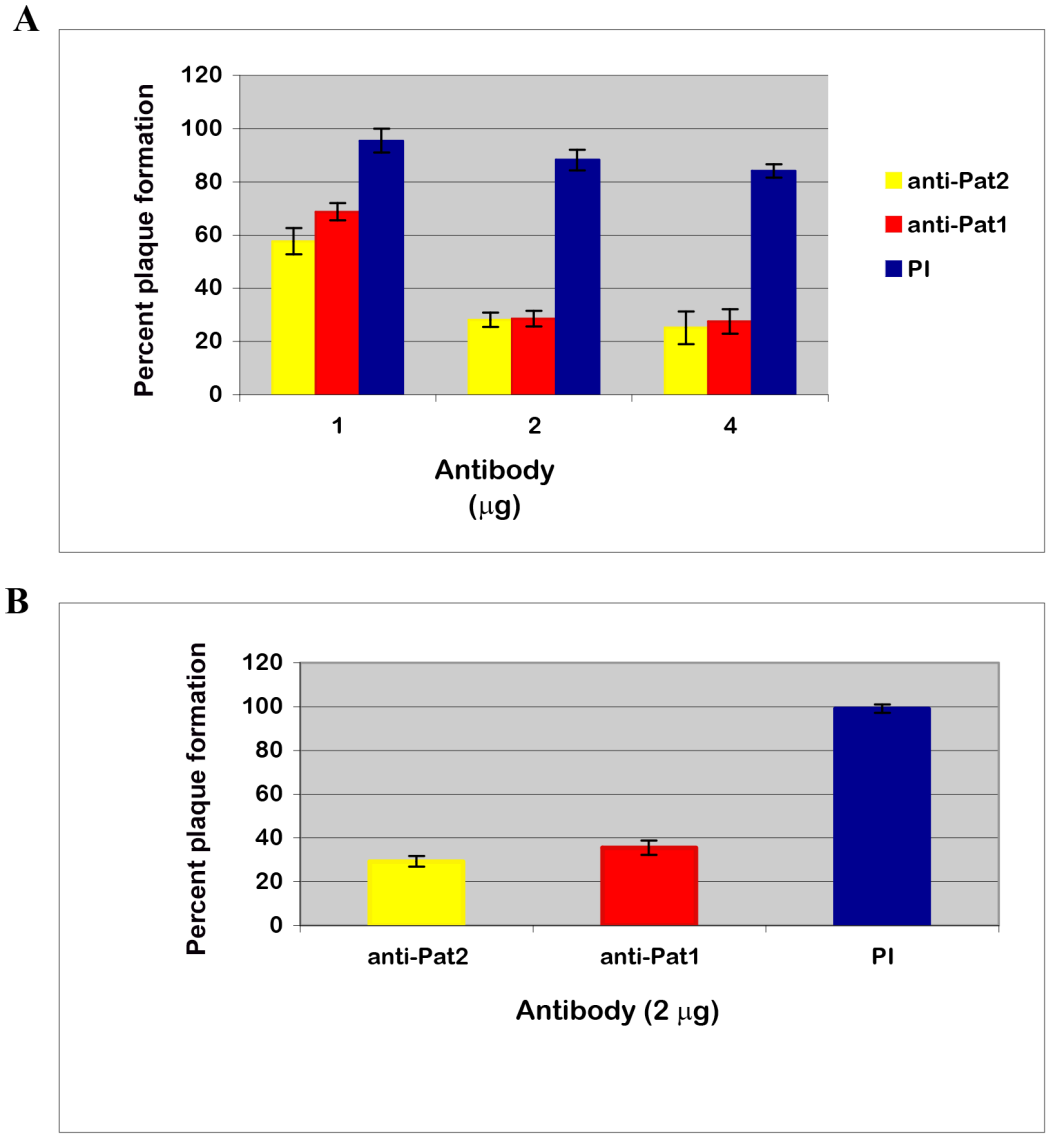
Effect of anti-Pat1 and anti-Pat2 pretreatment on *R. typhi* infection by plaque assay. Rickettsiae were treated with affinity purified anti-Pat1, anti-Pat2 or pre-immune IgG (PI) for 30 min on ice, followed by incubation for infection into Vero76 for 1 hour at 34°C and 5% CO_2_. *R. typhi* infected cells were left unwashed (**A**) or washed (**B**) before addition of agar for plaque assay as described (see Materials and Methods). The effect of antibody treatment on *R. typhi* was determined in percent (%) plaque formation by antibody treated rickettsiae with respect to that by no treatment. Error bars represent standard errors of the means. In both Panel **A** and **B**, percent (%) plaque formation by antibody pretreated *R. typhi* were found to be significantly different (P<0.05[two-tail t-test]) from that by PI.

We sought to determine the role of Pat1 and Pat2 in *R. typhi* infection of Vero76 cells by neutralizing their function using anti-Pat1 and anti-Pat2 antibodies. The viability of anti-Pat antibody-treated rickettsiae was quantitatively determined using a plaque assay. This analysis clearly demonstrated that the antibody-treated *R. typhi* decreased rickettsial infectivity and survival as compared to that by pre-immune IgG treated *R. typhi* (**Fig. 7A**). Plaque formation was significantly reduced in anti-Pat1 and anti-Pat2 pretreated groups as compared to the preimmune IgG treated group (**Fig. 7A**) and showed no apparent difference in plaque size for antibody treated rickettsiae with respect to preimmune IgG treated rickettsiae. Similarly, in a separate experiment, we removed excess antibodies and unattached rickettsiae from the host cells by washing prior to the plaque assay. These data showed that *R. typhi* plaques were decreased to 29.27%±2.33% or 35.49%±3.3% for anti-Pat2 or anti-Pat1 antibody, respectively, as compared to that by pre-immune IgG (**Fig. 7B**). The data also indicated a similar decrease in *R. typhi* plaque formation whether or not the excess antibody and unattached rickettsiae were removed before addition of agar for the plaque assay (**Fig. 7A, B**). Together, these data indicate that *R. typhi* Pat1 and Pat2 are involved at the early stage of *R. typhi* infection of host cells.

**Figure 8:**
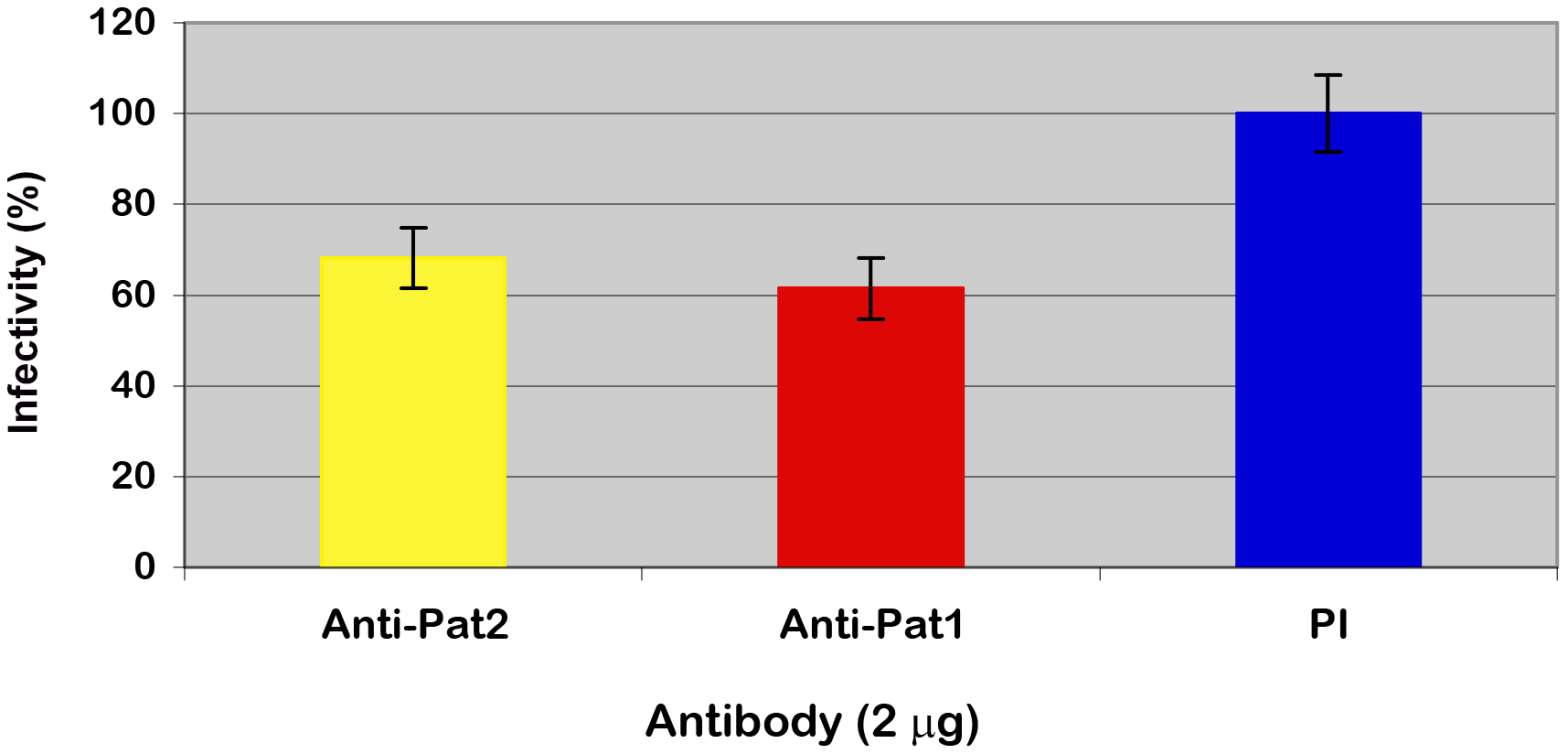
Effect of anti-Pat1 and anti-Pat2 antibody pretreatment on *R. typhi* infectivity of Vero76 cells by IFA. Rickettsiae were treated with 2 µg of affinity purified anti-Pat1, anti-Pat2 or pre-immune IgG (PI) for 30 min on ice, followed by incubation for infection of Vero76 for 18 hour at 34°C and 5% CO_2_. The infected cells were labeled for IFA and % infectivity was determined as described (see Materials and Methods). Infectivity (%) was determined in % host cells infected by *R. typhi* pretreated with anti-Pat1 or anti-Pat2 antibody with respect to that with PI. Error bars represent standard errors of the means. Infectivity (%) of anti-Pat1 or anti-Pat2 antibody pretreated *R. typhi* was found to be significantly different (P<0.05[two-tail t-test]) from that of PI.

Furthermore, an indirect immunofluorescent antibody assay was used to demonstrate the involvement of Pat1 and Pat2 in *R. typhi* infection of host cells via counting the percent of host cells infected by *R. typhi* pretreated with anti-Pat1 or anti-Pat2 with respect to those treated with preimmune IgG (See Materials and Methods). These data showed the significant decrease in infection of Vero76 cells by *R. typhi* pretreated with anti-Pat1 or anti-Pat2 antibodies as determined by IFA (**Fig. 8**). Thus, the IFA results suggest that pretreatment of *R. typhi* with anti-Pat2 or anti-Pat1 antibody blocks rickettsial invasion of host cells, confirming the role of Pat1 and Pat2 during the early stage of *R. typhi* infection of host cells.

**Figure 9:**
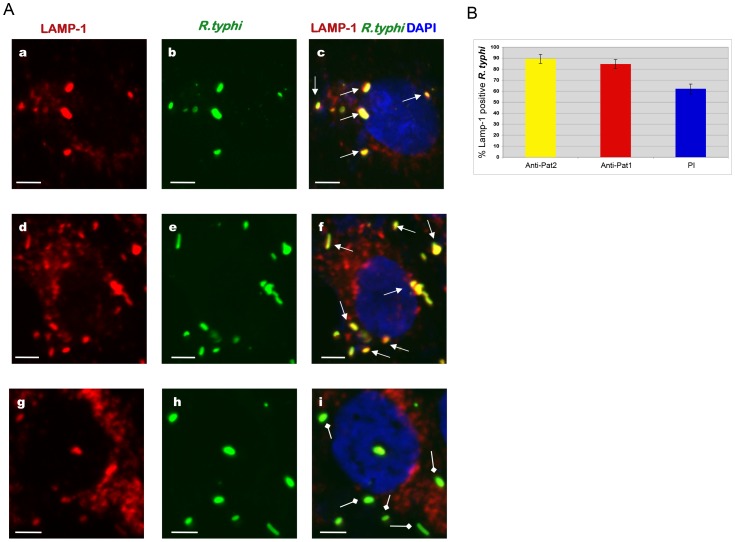
Effect of anti-Pat1 and anti-Pat2 pretreatment on *R. typhi* phagosome escape by IFA. Rickettsiae were incubated with 2 µg of affinity purified anti-Pat1, anti-Pat2 or pre-immune IgG (PI) for 30 min on ice. Pretreated rickettsiae were added onto Vero76 monolayer followed by incubation at 34°C and 5% CO_2_. At 30 min postinfection, the cells were fixed with 4% paraformaldehyde and immunolabeled with anti-LAMP-1 (at 1∶100 dilution) mouse monoclonal antibodies (Abcam Inc) and anti-*R. typhi* rat serum (at 1∶500 dilution) as primary antibodies. The anti-rat-Alexa Fluor-488 (green) and anti-mouse-Alexa Fluor-594 (red) were used as secondary antibodies. The cell nuclei were stained with DAPI (blue). Samples were viewed under a LSM5DUO confocal microscope and images were processed using ZEN imaging and analysis software. (**A**) Representative micrograph of anti-Pat1 (panels a to c), anti-Pat2 (panels d to f) or pre-immune IgG (panels g to i) treated rickettsiae that were infected of Vero76 cells. The white arrows show rickettsiae in LAMP-1 positive phagosome, with the square head arrow showing rickettsiae escaping or escaped from the phagosome. Scale Bar = 5 µm. (**B**) Quantitation of percent rickettsiae in LAMP-1 positive phagosome was determined as percent LAMP-1 positive *R. typhi* by scoring 100 bacteria for each treatment. Each treatment was repeated four times. Error bars represent standard error of the means. Percent LAMP-1 positive *R.typhi* for anti-Pat1 or anti-Pat2 pretreatment was found to be significantly different (P<0.05[two-tail t-test]) from that of PI.

Long hypothesized to be mediated by PLA activity [16], rickettsial phagosome escape was previously shown to occur between 30 to 50 min postinfection [36]. Accordingly, we examined the role of Pat1 and Pat2 in *R. typhi* phagosome escape into the host cytoplasm during the early stage of infection. Vero76 cells were infected with *R. typhi* pretreated with anti-Pat1, anti-Pat2 or preimmune IgG. At 30 min postinfection, rickettsial phagosome escape was monitored by IFA using the endosomal/lysosomal marker lysosomal-associated membrane protein 1 (LAMP-1). We observed that *R. typhi* pretreated with anti-Pat1 (**Fig. 9A**, panels a to c) or anti-Pat2 (**Fig. 9A**, panels d to f) antibodies mostly colocalized with the LAMP-1 marker, suggesting the rickettsiae are still enclosed in the phagosome at 30 min postinfection. However, *R. typhi* pretreated with preimmune IgG are relatively less LAMP-1 positive at 30 min postinfection (**Fig. 9A**, panels g to i), supporting rickettsial escape from phagosome. Further quantification revealed that the percentage of *R. typhi* in LAMP-1 positive phagosome was significantly higher for rickettsiae pretreated with anti-Pat1 or anti-Pat2 antibody compared to that with pre-immune IgG (**Fig. 9B**). Together, these data suggest that antibody neutralization of Pat1 or Pat2 prior to host cell infection blocks or delays *R. typhi* phagosome escape.

## Discussion

In an earlier report, we showed that *pat2* (*RT0522*), but not *pat1* (*RT0590*), is transcribed at all stages of *R. typhi* intracellular growth in Vero76 cells [19]. This led to our characterization of Pat2 as a functional patatin that is secreted into the host cytoplasm during rickettsial infection. However, the presence of *pat1* in all sequenced *Rickettsia* genomes, coupled with the pseudogenization of *pat2* in 76% of these genomes, prompted us to delve deeper at a possible role for the ubiquitous Pat1 in rickettsial biology. In this study, we showed the expression of Pat1 protein in *R. typhi* cultured in Vero76 cells, despite the inability to detect *pat1* mRNA [19] under the same experimental conditions. This phenomenon is perhaps best explained by a potential difference in lifespan for *pat1* mRNA and protein. For example, some studies have shown that a lack of correlation between mRNA and protein abundance can be attributed to longer half-lives of protein (longer than cell cycle) relative to mRNA (a few minutes) in some prokaryotes [37], [38]. Importantly, labile transcripts relative to protein lifespan may be particularly common for genes activated infrequently relative to workhorse genes (e.g., replication and translational machinery genes, basic metabolic genes, etc.), resulting in gross underestimation of gene/protein function in studies based solely on transcript (and not protein) detection.

The present results supporting the function of Pat1 in rickettsial biology are corroborated by the ubiquity of the *pat1* gene in 46 *Rickettsia* genomes, which include species and strains ranging from pathogenic to non-pathogenic (**Fig. 1B**). The presence in the rickettsial mobilome of plasmid-encoded *pat1* genes, and their likely recombination with chromosomal *pat1* genes multiple times throughout *Rickettsia* evolution, strongly imply an essential function for Pat1 in the rickettsial intracellular life cycle. Phylogeny estimation grouped rickettsial Pat1 proteins in a clade with other bacteria possessing an intracellular lifestyle, including *Coxiella burnetii*, “*Candidatus* Odyssella thessalonicensis”, “*Candidatus* Amoebophilus asiaticus”, and Rickettsiales endosymbiont of *Trichoplax adhaerens* (**Fig. S1**). The latter three genomes encode multiple Pat1 homologs, with an extraordinary array of patatins encoded in the “*Candidatus* Amoebophilus asiaticus” genome [39]. Other closely related clades also contained bacteria with an intracellular lifestyle, especially members of Bacteroidetes. This implies that PNPLA8, PNPLA9, and Pat17 patatin-like phospholipases may be common proteins shared by intracellular bacteria, orchestrating functions such as the internalization process, phagosomal escape, and host cell lysis for cell-to-cell spread. Importantly, our phylogeny estimations for both Pat1 and Pat2 proteins suggests a substantial degree of lateral gene transfer (LGT) responsible for the origin of Pat genes in *Rickettsia* spp. and other bacteria, a result consistent with the origin of PLA_2_s in *P. aeruginosa* (ExoU) [40] and Group A *Streptococcus* (SlaA) [41] via LGT.

Our prior study determined that *R. typhi* Pat2 is secreted into the host cytoplasm during infection, a result consistent with a bioinformatics-based prediction of Pat2 as a secreted protein [19]. However, Pat2 is not predicted to encode an N-terminal signal peptide sequence, suggesting the involvement of a secretion pathway outside of Sec-dependent translocation. Similarly to Pat2, our bioinformatics analysis [42], [43], [44] predicts Pat1 to be noncytoplasmic with no Sec-dependent signal peptide sequence. In this report, by IFA, we have shown that both Pat1 and Pat2 are co-localized with *R. typhi*, and also form punctate structures throughout the host cell cytoplasm, indicating the translocation of Pat1 and Pat2 from rickettsiae into the host cytoplasm. Data from immunoblotting analysis also revealed the translocation of Pat2 (positive control) and Pat1 into the host cytoplasm. However, the slightly slower mobility of rickettsiae-associated Pat1 and Pat2 proteins versus Pat1 and Pat2 proteins that are translocated into the host cytoplasm might be due to the cleavage of a short signal sequence during translocation. While currently unknown, a potential processing mechanism (cleavage or modification) for Pat1 and Pat2 during or after translocation from the rickettsial cell will likely emerge via the identification of the apposite secretion pathway(s) for these proteins.

Insight into the secretion pathway responsible for Pat1 and Pat2 delivery to host cells comes from consideration of other secreted bacterial Pats. The highly cytotoxic ExoU (*P. aeruginosa*) is secreted via the type III secretion system (T3SS) [23], [45], while the VipD Pat of *Legionella* spp. is secreted via the I-like (Dot/Icm) T4SS [46]. *Rickettsia* spp. do not encode a T3SS, but do encode a P-like T4SS termed the Rickettsiales *vir* homolog (*rvh*) T4SS [47], [48]. Substrates translocated by both I- and P-like T4SSs often contain C-terminal signals that mediate their translocation out of the bacterial cytoplasm and into the extracellular milieu (or host cytoplasm) [49], [50], [51], [52]. The conserved nature of the C-terminal sequences of both Pat1 and Pat2, coupled with our detection of sites evolving under positive selection within the T region of Pat1 (**Fig. 2B**), suggest these proteins may be *rvh* T4SS substrates. Alternatively, T1SS substrates also possess C-terminal secretion signals [53], and *Rickettsia* spp. encode the OM pore component of T1SSs (TolC) and a series of membrane fusion and ABC transporter protein pairs that probably all assemble as T1SSs [26]. While the T1SS in *Rickettsia* spp. has not been demonstrated to secrete protein, protein substrates from the closely related *Ehrlichia chaffeensis* (Rickettsiales: Anaplasmataceae) have recently been demonstrated to be secreted via the T1SS [54]. Additionally, our very recent work suggests that an ankyrin domain-containing protein of *Rickettsia* spp. is exported extracellularly via a non-canonical secretion pathway that utilizes both the Sec translocon and TolC, suggesting that TolC has a function in *Rickettsia* protein secretion [55]. Accordingly, in the future we will investigate both the *rvh* T4SS and the T1SS for putative roles in Pat1 and Pat2 secretion.

Both ExoU of *P. aeruginosa* [23], [24] and Pat2 of *R. typhi* [19] have been reported to be cytotoxic to mammalian and yeast cells, and their PLA activity is required for cytotoxicity. Our data show that the expression of *R. typhi* Pat1 is also cytotoxic in yeast cells, requiring Ser/Asp catalytic sites for PLA activity and cytotoxicity. Our current data on Pat1 and previously reported data on Pat2 [19] show that the cytotoxicity and PLA activity of these two *R. typhi* enzymes are relatively low compared to that of ExoU. These data support our earlier interpretation [19] that the low PLA activity is required to support obligate intracellular growth of *R. typhi* in host cells without inflicting any rapid damage (at least until bacteria lyse host cells to promote cell to cell spread). Importantly, the minimal (*pat2*) and deficient (*pat1*) detection of *R. typhi* Pat transcripts correlates with a milder cytotoxic phenotype of Pat1 and Pat2 relative to the highly potent ExoU.

The relatively higher cytotoxic effect and PLA activity of Pat2 [19] compared to Pat1 reported here suggest that Pat1 and Pat2 might be interacting with host cells differently. As discussed above, Pat1 and related PNPLA8, PNPLA9, and Pat17 patatin-like phospholipases, are encoded by many intracellular bacteria, not all of which are pathogens. These enzymes may have less of a cytotoxic effect on host cells if they are associated with functions that benefit the bacteria yet do not substantially impact the fitness of the host. Alternatively, the closest homologs of Pat2 that have been functionally characterized, namely ExoU (*Pseudomonas* spp.) and VipD (*Legionella* spp.), are bona fide virulence factors. The potency of ExoU is demonstrated by its destruction of cellular membranes of mammalian cells, which causes rapid necrotic death [23], as well as its role in the killing of environmental amoeba [56]. Induced during stationary phase, the most virulent stage of the *L. pneumophila* life cycle, VipD interferes with vesicular trafficking and promotes intracellular growth [46], [57]. Thus, it is reasonable to suggest that Pat2 may have a function that is more detrimental to host cells than Pat1, possibly in the lysis of host cells for cell-to-cell spread.

Dependence on eukaryotic activator(s) for PLA_2_ activity has been reported for *P. aeruginosa* ExoU [23], [30], and it has been shown that certain preparations of Cu^2+^, Zn^2+^-SOD1 act as a cofactor to activate ExoU phospholipase activity [33]. A recent report on the Pat2 homolog RP534 of *R. prowazekii* demonstrated the stimulation of RP534 PLA activity by SOD1 [20]. Our current data on Pat1 and our previous report on Pat2 [19] demonstrate their PLA activities in the presence of Vero76 cell lysate. Our further analysis of *R. typhi* Pat1 and Pat2 proteins reveal that bovine liver SOD1 activates their PLA activities. The level of activation by bovine liver SOD1 is similar to that by Vero76 cell lysate, indicating the similarity in the enzymatic activation for bacterial patatin-like phospholipases. A recent report by Anderson et al [58] on enzymatic activation of ExoU showed that several ubiquitin isoforms, as well as a subpopulation of ubiquitylated SOD1 and ubiquitylated mammalian proteins, activate ExoU. Although, we have shown here that *R. typhi* Pat1 and Pat2 proteins required eukaryotic activator(s) (Vero76 cell lysate or bovine liver SOD1) for their PLA activities, further analysis of enzymatic activator for Pat1 and Pat2 will be addressed in our future work.

The role of PLA_2_ has long been hypothesized to be involved in rickettsial infection of host cells and phagosome escape [15], [16]; however, this enzymatic activity has never been demonstrated within the known rickettsial proteome. In this report, we have shown by IFA that pretreatment of *R. typhi* with anti-Pat1 or anti-Pat2 antibodies negatively affects rickettsial phagosome escape. Furthermore, we have demonstrated by plaque assay and IFA that pretreatment of *R. typhi* with anti-Pat1 or anti-Pat2 antibodies significantly decreases *R. typhi* infectivity into Vero76 cells. However, the effect of anti-Pat1 or anti-Pat2 pretreatment on *R. typhi* infectivity into Vero76 cells is relatively low as determined by IFA compare to that by plaque assay. This disparity in *R. typhi* infectivity might have resulted due to the difference in our assays for *R. typhi* infectivity into Vero76 cells. By IFA, we measured the total *R. typhi* infected host cells, resulting in higher infectivity compared to that by plaque assay. However, by plaque assay, we measured the number of plaques produced on host lawn by only plaque-forming *R. typhi*, resulting in lower infectivity compared to that by IFA. Together these data suggests that both Pat1 and Pat2 are rickettsial surface exposed, and neutralization of Pat1 or Pat2 by their respective antibody blocks *R. typhi* infection of host cells and also blocks or delays rickettsial phagosome escape. This supports the long-standing hypothesis that PLA_2_ proteins are involved in early stage of rickettsial infection.

In this manuscript, as well as our previous report [19], we have shown that Pat1 and Pat2 are translocated into the host cytoplasm during intracellular growth. In another report from our laboratory, we have shown the detection of Pat2 on *R. typhi* surface by experimentally surface-labeling with thiol-cleavable sulfo-NHS-SS-biotin and neutravidin affinity purification of labeled proteins, followed by identification by LC-MS/MS [27]. Together, these data imply that Pat1 and Pat2 are rickettsial surface exposed proteins; however, during intracellular growth, both are translocated from rickettsiae into the host cytoplasm. Additionally, here we have demonstrated that pretreatment of *R. typhi* by anti-Pat1 or anti-Pat2 antibody blocked *R. typhi* infection of host cells and also blocked or delayed rickettsial phagosome escape. The PLA functional assay data also revealed that *R. typhi* Pat1 and Pat2 proteins required eukaryotic activator(s) for their PLA activities. Collectively, these results suggest that surface exposed Pat1 or Pat2 of extracellular *R. typhi* are enzymatically inactive without eukaryotic activator(s), but are involved in rickettsial adherence or internalization into host cells at the early stage of infection. However, pretreatment of surface exposed Pat1 or Pat2 of extracellular *R. typhi* with their respective antibody neutralizes these proteins before they can engage eukaryotic host factors for their enzymatic activation and subsequent biological activity inside host cells.

The results presented in this communication extend our study of the functional characterization of *R. typhi* PLA proteins Pat1 and Pat2. Our future research will determine the secretion pathway(s) for Pat1 and Pat2 from the bacterial cytoplasm, mechanism of translocation into the host cytoplasm during intracellular growth, host subcellular localization and molecular targets, mechanism of activation for PLA activity, and the overall roles of these patatins in the *R. typhi* intracellular life cycle, especially regarding the early stage of infection including phagosomal escape and host cell lysis.

## Materials and Methods

### Bioinformatics analysis

Orthologs of *R. typhi* Pat1 (YP_067537) and Pat2 (YP_067473) were extracted from the PATRIC web site [59]. All sequences included in the analyses are listed in **Table S1**. Identical sequences across more than one rickettsial genome were culled for redundancy. Orthologous groups for Pat 1 (*n* = 33) and Pat2 (*n* = 11) were aligned using MUSCLE v3.6 [60], [61] with default parameters. For both datasets, the program DIVEIN [62] was used to estimate percent protein divergence using the WAG amino acid substitution model. Alignments were screened for the presence of signal sequences using SignalP v.4.0 [63], LipoP v.1.0 [43] and Phobius [64]. Potential transmembrane spanning regions were predicted using the transmembrane hidden Markov model (TMHMM) v.2.0 [65]. Both alignments were scanned for repeats using HHrepID [66]. The chromosomal location of both *pat1* and *pat2* genes across 46 *Rickettsia* genomes was assessed using the Compare Region Viewer at PATRIC.

To gain an understanding of the evolutionary origin of rickettsial genes encoding Pat1 and Pat2, datasets were constructed to estimate patatin phylogeny across diverse prokaryotic and eukaryotic lineages. Using *R. typhi* Pat1 and Pat2 as queries, BLASTP searches were performed against the nr (All GenBank+RefSeq Nucleotides+EMBL+DDBJ+PDB) database, coupled with a search against the Conserved Domains Database [67]. Default matrix parameters (BLOSUM62) and gap costs (Existence: 11 Extension: 1) were implemented, with an inclusion threshold of 0.005. Searches, with composition-based statistics and no filter, were performed within the following five databases: 1) “Rickettsiales”, 2) “*Alphaproteobacteria* (minus Rickettsiales)”, 3) “*Proteobacteria* (minus *Alphaproteobacteria*)”, 4) “Bacteria (minus *Proteobacteria*)”, and 5) “minus Bacteria”. The top 20 (query-dependent) subjects from each search resulting in significant (>40 bits) alignments were retrieved, compiled and aligned using MUSCLE v3.6 (default parameters). Ambiguously aligned positions were culled using Gblocks [68], [69]. Phylogenies were estimated for both Pat1 (*n* = 143) and Pat2 (*n* = 93) datasets under maximum likelihood using RAxML v.7.2.8 [70]. A gamma model of rate heterogeneity was used with estimation of the proportion of invariable sites. Branch support was assessed with 1000 bootstrap pseudoreplications.

Rickettsial Pat1 and Pat2 proteins were evaluated to determine if any regions of these molecules are evolving under positive selection. Using the EMBOSS tranalign tool (http://emboss.bioinformatics.nl/cgi-bin/emboss/tranalign), protein sequence alignments were used as templates to align the corresponding nucleotide sequences according to respective codons. The ratios of non-synonymous to synonymous substitutions across all nucleotide alignments were calculated using Selecton v2.4 [71].

### Bacterial strain and host cell

*R. typhi* strain Wilmington (ATCC: VR-144) was propagated in Vero76 (African green monkey kidney, ATCC: CRL-1587) cells. The host Vero76 cells were grown in DMEM (Dulbecco's modification of Eagle's medium with 4.5 gram/liter glucose and L-glutamine, Mediatech, Inc., Manassas, VA) supplemented with 5% fetal bovine serum (Mediatech, Inc., Manassas, VA) at 37°C and 5% CO_2_ in air atmosphere.

### Extraction of genomic DNA

Rickettsiae were propagated and partial purified from Vero76 cells as previously described [12]. Genomic DNA of *R. typhi* was extracted using the Wizard genomic DNA purification kit (Promega, Madison, WI).

### Cytotoxicity assay in yeast

The *R. typhi RT0590 (pat1)* gene was cloned by PCR into the *KpnI* and *XhoI* sites of yeast expression vector pYES2/CT with C-terminal epitope (V5/6× His) tags (Invitrogen-Life Technologies, Carlsbad, CA) according to manufacturer's instruction, using primers AZ5362 and AZ5363 (**Table 1**). The constructed plasmid pYES-590 (Pat1-wt) was confirmed by sequencing. The sequence of the *RT0590* gene and deduced amino acid sequence from *R. typhi* were analyzed using MacVector 7.1.1 software (Genetic Computer Group, Inc. Madison, WI). Sequence comparisons were performed with BLAST tools at PATRIC and NCBI.

**Table 1:** Primers used in this study.

Primer	Sequence (5′ to 3′) a ^, ^ b
AZ5362	ggt acc ATG GTA GAT ATA AGC AAT ACT
AZ5363	ctc gag AGC TAC AAT TTC ATT ATA ACC AGG
AZ6156	GGT ACC ATG GTG GAT ATA AGC AAC
AZ6157	CTC GAG CGC CAC GAT CTC ATT ATA
FR1008	GGC GGC ACC GCT GTC GGA GGC CTT
FR1009	ACT ATT ATC GCC GGG GGG ATC TAC

a The nucleotides incorporated to generate restriction sites are indicated in lower case.

b Primers designed based on *R. typhi* str. Wilmington genome sequence (NCBI acc. no. NC_006142).

The coding sequence of the *RT0590* gene was codon-optimized (*RT0590HS*) to mammalian cells by BlueHeron Biotechnology, Bothell, WA. The *RT0590HS* sequence was cloned into the *KpnI* and *XhoI* sites of pYES2/CT vector with C-terminal epitope tags. The constructed plasmid pYES-590HS (Pat1-co) was confirmed as described above.

Mutation at catalytic sites (Ser-51 and Asp-199) of RT0590 was introduced in plasmid pYES-590HS using the QuickChange Lightning Multi Site-Directed Mutagenesis kit (Stratagene) according to manufacturer's instruction. The primer used for site directed mutagenesis of Ser-51 to Ala is FR1008, and that for Asp-199 to Ala is FR1009 (**Table 1**). The constructed plasmid pYES-590SD (Pat1-SD) was confirmed by sequencing as described above.

*Saccharomyces cerevisiae* strain INVSc1 (Invitrogen) was transformed with the constructed plasmids using the Frozen-EZ Yeast transformation kit (ZYMO Research, Orange, CA) following manufacturer's protocol. The yeast transformants were assayed for cytotoxicity by colony forming unit (CFU) assay as previously described [19]. Briefly, for CFU assay, the yeast transformants were serially diluted in SC-U without a carbon source and plated on inducing (SC-U+Gal) and repressing (SC-U+Glu) agar. After incubation at 30°C for 3 days, the colonies were counted to determine the %CFU on inducing agar with respect to repressing agar.

### Expression and purification of recombinant proteins for PLA_2_ assay

The coding sequence of *RT0590HS* was subcloned by PCR from pYES-590HS plasmid into pTrcHis2TOPO-TA vector (Invitrogen) for expression in *Escherichia coli* using primer pair AZ6156 and AZ6157 (**Table 1**) to generate the plasmid pTrc-590HS for the production of recombinant protein Pat1 (RT0590). Mutations at catalytic sites (Ser-51 and Asp-199) of RT0590 (Pat1) were introduced in plasmid pTrc-590HS using the procedure described above to generate the plasmid pTrc-590SD for the production of recombinant mutant protein Pat1-SD (RT0590SD).

*E. coli* TOP10 chemically competent cells (Invitrogen) were transformed with pTrc-590HS (Pat1), pTrc-590SD (Pat1-SD) and pTrc-522HS (Pat2, as positive control) [19] according to the manufacturer's instructions. The recombinant proteins with C-terminal 6×His tag were expressed in TOP10 cells as previously described [19] and purified by Ni_NTA magnetic agarose beads (Qiagen) following manufacturer's protocol. The elution buffer of the purified recombinant proteins was exchanged to PLA_2_ Assay buffer (50 mM Tris-HCl, 100 mM NaCl, 1 mM CaCl2, pH 8.9) using Amicon Ultracel-50k, 50,000 MWCO (Millipore, Billerica, MA). The concentration of purified proteins was determined by BCA protein assay kit (Pierce, Rockford, IL), with purity assessed by Imperial Protein stained gel (**Fig. S3**). The identity of the purified recombinant protein RT0590 was further confirmed by mass spectrometry (MS) analyses at the protein core facility, Center for Vascular and Inflammatory Diseases, University of Maryland, Baltimore, MD.

### Phospholipase activity assay

The PLA_2_ activities of recombinant proteins were measured by using Fluorogenic phospholipid substrates (specific to PLA_2_ enzyme activity): 1-*O*-(6-BODIPY 558/568-aminohexyl)-2-BODIPY FL C_5_-*sn*-glycero-3-phosphocholine (Red/Green BODIPY PC-A2) (Invitrogen) according to the manufacturer's instruction [19]. Briefly, the cleavage of the *sn-2* bond of Red/Green BODIPY PC-A2 (Fluorogenic phospholipid substrates) by the PLA_2_ enzyme results in an increase of fluorescence emission. The Red/Green BODIPY PC-A2 substrate was suspended to a final concentration of 1 mM in dimethyl sulfoxide. The substrate-liposome mix was prepared by mixing 25 µl of 1 mM Red/Green BODIPY PC-A2 substrate, 25 µl of 10 mM of 1,2-dioleoyl-*sn*-glycero-3-phosphocholine (DOPC) and 25 µl of 1,2-dioleoyl-*sn*-glycero-3-phospho-(1′-*rac*-glycerol) (sodium salt) (DOPG) in 5 ml of PLA_2_ assay buffer. Substrate-liposome mix (50 µl) was added to 50 µl of PLA_2_ samples containing recombinant proteins and eukaryotic activator(s) [either Vero76 cell lysate or bovine liver superoxide dismutase (SOD) at 0.5 µg/µl]. For PLA_2_ inhibitor assays, MAPF was added to the reaction at a final concentration of 0.5 µM. The reaction mixture was incubated at room temperature for 60 minutes. The fluorescence emission was measured using a FLUOstar Omega plate reader (BMG Labtech, Germany) for excitation at 485 nm and emission at 520 nm. The phospholipase A (PLA) activity of recombinant protein was calculated following manufacturer instruction as fluorescent emission(Emission_Sample_ – Emission_EukaryoticCofactor_ – Emission_Blank_)/recombinant protein(µg)/minute.

### Antibodies

The preparation and affinity purification of rabbit anti-Pat2 and anti-EF-Ts (Elongation factor Ts) antibodies were executed as previously described [19]. The rabbit anti-Pat1 antibody was generated and affinity purified by PrimmBiotech Inc (Cambridge, MA) against the first (N-terminal) 305 aa of *R. conorii* Pat1 (RC0922). This sequence contains all of the conserved motifs required for PLA_2_ activity (the glycine-rich, serine hydrolase and active site aspartate) [19] and has 83% aa identity with the corresponding region of *R. typhi* Pat1 (RT0590).

### Translocation assay by western blotting

Vero 76 cells, either infected or uninfected with *R. typhi*, were incubated in culture medium at 34°C and 5% CO_2_ for 48 hours. The cells were washed in cold PBS and lysed in cold lysis buffer (0.1% Triton X-100 in PBS supplemented with complete Mini EDTA-free protease inhibitor) by incubating on ice [72]. The disrupted cells were centrifuged at 16000×g for 10 min at 4°C to separate the supernatant containing rickettsial secreted proteins and host soluble proteins from the pellet containing host debris with intact rickettsiae. The supernatant was filtered through a 0.45 µm pore-size filter (Millipore) and proteins were precipitated by 1/10 volume of trichloroacetic acid and 1/100 volume of 2% sodium deoxycholate for overnight at 4°C.

Samples from the pellet and supernatant were separated on a 4 to 20% Tris-glycine precast gel (Invitrogen) with 1× Tris-glycine-SDS running buffer (BioRad), and subsequently transferred to a PVDF membrane as previously described [19]. Briefly, the membrane was probed with rabbit anti-Pat1 abs, rabbit anti-Pat2 abs (as positive control for *R. typhi* secreted protein), rabbit anti-rOmpB antibody (as control for *R. typhi* surface protein) [27] or rabbit anti-EF-Ts abs (as control for *R. typhi* cytoplasmic protein). As a host cytoplasmic protein control, membranes were probed with a mouse monoclonal antibody against glyceraldehydes 3 phosphate dehydrogenase (GAPDH) (Abcam Inc., Cambridge, MA).

### Translocation assay by immunofluorescence labeling

Partially purified rickettsiae were added onto monolayer of Vero76 cells for infection at 34°C and 5% CO_2_. At 24 hr postinfection, the infected cells were washed three times with PBS and fixed with 4% paraformaldehyde (in PBS) at room temperature for 20 min. The cells were washed three times with PBS and permeabilized in permeabilization buffer (1.0% BSA, 0.1% saponin in PBS) before immunofluorescence labeling. For immunofluorescence staining, cells were immunolabeled with anti-Pat1 or anti-Pat2 rabbit antibodies (at 1∶200 dilution in permeabilization buffer) and anti-*R. typhi* rat serum (at 1∶500 dilution in permeabilization buffer) as primary antibodies. *R. typhi* infected Vero76 cells were also labeled similarly with rabbit pre-immune serum (at 1∶200 dilution in permeabilization buffer) and anti-*R. typhi* rat serum (at 1∶500 dilution in permeabilization buffer). The anti-rat-Alexa Fluor-594 (red) and anti-rabbit-Alexa Fluor-488 (green) were used as secondary antibodies. The cell nuclei were stained with DAPI (blue). Samples were viewed under a LSM5DUO confocal microscope at the core imaging facility, Department of Physiology, University of Maryland Baltimore and images were processed using ZEN imaging and analysis software.

### Effect of anti-Pat1 and anti-Pat2 antibodies on *R. typhi* infection of Vero76 cells

#### Plaque assay

Rickettsiae were partially purified from *R. typhi* infected (>90%) Vero76 cells as follows. The infected cells were harvested in 5 ml of DMEM supplemented with 5% FBS using cell scraper. Rickettsiae were released from host cells by mild sonication for 15 seconds using a sonic dismembrator (Fisher Scientific, PA). The disrupted host cells were centrifuged at 1,000×*g* for 10 min at 4°C to remove host cell debris or any remaining intact host cells. The supernatant was filtered through a 5 µm-pore-size filter (Millipore). The filtrate (100 µl), containing partially purified rickettsiae, was incubated with affinity purified anti-Pat1, anti-Pat2 or pre-immune IgG in an ice bath for 30 min. The antibody pretreated rickettsiae were added to a monolayer of Vero76 cells for infection and incubated for 1 hour at 34°C and 5% CO_2_. Following incubation, one set of infected cells were washed with DMEM medium supplemented with 5% FBS, with a second set of infected cells left unwashed. For both sets of infected cells, DMEM medium supplemented with 5% FBS and 0.5% agarose was added and incubated at 34°C and 5% CO_2_. Ten days later, plaques were stained overnight with 0.01% neutral red. The number of plaques for each treatment was counted to determine the effect of antibodies on *R. typhi* infection of host cells and the data were presented as percent (%) plaque formation by antibody treated rickettsiae with respect to that by no treatment. Each experiment was repeated at least four times.

#### Immunofluorescence assay (IFA)

Rickettsiae were partially purified and treated with 2 µg affinity purified anti-Pat1, anti-Pat2 or pre-immune IgG as mentioned above. The pretreated rickettsiae were added to a monolayer of Vero76 cells for infection and incubated for 18 hour at 34°C and 5% CO_2_. The infected cells were washed three times with PBS and fixed with 4% paraformaldehyde (in PBS) at room temperature for 20 min. The cells were washed three times with PBS before immunofluorescence labeling. For immunofluorescence staining of extracellular rickettsiae, infected Vero76 cells were incubated with rat anti-*R. typhi* serum in PBS, 1.0% BSA for 45 min at room temperature and then incubated with donkey anti-rat alexa fluor 488-conjugated IgG (Molecular Probes) in PBS, 1.0% BSA for 30 min at room temperature. In order to stain total rickettsiae, infected cells were permeabilized for 10 min in permeabilization buffer (1.0% BSA, 0.1% saponin in PBS). The cells were re-incubated with rat anti-*R. typhi* serum in permeabilization buffer for 45 min. at room temperature and with donkey anti-rat alexa fluor 594-conjugated IgG (Molecular Probes) in permeabilization buffer for 30 min. The cells were washed with PBS and mounted with Vectashield mounting medium containing 4′,6-diamidino-2-phenylindole (DAPI; Vector Laboratories, CA). Samples were viewed under a Nikon Eclipse E600 fluorescence microscope to enumerate the number of host nuclei, extracellular and total rickettsiae. The number of host cells infected with rickettsiae was counted to determine the effect of antibody treatment on *R. typhi* invasion. Approximately 100 host cells were enumerated for each antibody treatment for *R. typhi* invasion assay. Each experiment was repeated four times.

Rickettsiae were treated with 2 µg of affinity purified anti-Pat1, anti-Pat2 or pre-immune IgG for 30 min on ice as mentioned above. Pretreated rickettsiae were added onto Vero76 monolayer followed by centrifugation at 200×g for 5 min at room temperature to induce contact and incubation at 34°C and 5% CO_2_. At 30 min postinfection, the cells were washed three times with PBS and fixed with 4% paraformaldehyde (in PBS) at room temperature for 20 min. The cells were washed three times with PBS before immunofluorescence labeling. The cells were immunolabeled with anti-LAMP-1 (at 1∶100 dilution) mouse monoclonal antibodies (Abcam Inc) and anti-*R. typhi* rat serum (at 1∶500 dilution) as primary antibodies. The anti-rat-Alexa Fluor-488 (green) and anti-mouse-Alexa Fluor-594 (red) were used as secondary antibodies. The cell nuclei are stained with DAPI (blue). Samples were viewed under a LSM5DUO confocal microscope and images were processed using ZEN imaging and analysis software. The number of rickettsiae in LAMP-1 positive phagosome was determined by scoring 100 bacteria for each treatment. Each treatment was repeated four times.

### Accession numbers not listed in the respective figure legends are as follows

YP_538013, YP_538012, YP_538517: *Rickettsia bellii* RML369-C; YP_001495950, YP_001495691: *Rickettsia bellii* OSU 85-389; YP_001492093: *Rickettsia canadensis* McKiel; YP_005299400: *Rickettsia canadensis* CA410; VBIRicHel217856_0676, VBIRicHel217856_0986: *Rickettsia helvetica* C9P9; YP_246376, YP_247427: *Rickettsia felis* URRWXCal2; YP_005414840: *Rickettsia australis* Cutlack; YP_001493712: *Rickettsia akari* Hartford; YP_067537, YP_067473: *Rickettsia typhi* Wilmington; YP_005405102, YP_005413959: *Rickettsia prowazekii* GvV257; NP_220970, NP_220907: *Rickettsia prowazekii* Madrid E; ZP_04699958: *Rickettsia* endosymbiont of *Ixodes scapularis*; YP_005365687, YP_005353138, YP_005365402: “*Candidatus* Rickettsia amblyommii” GAT-30V; YP_005390723, YP_005390510: *Rickettsia rhipicephali* 3–7-female6-CWPP; YP_001499568, YP_001499395: *Rickettsia massiliae* MTU5; YP_005301963, YP_005302159: *Rickettsia massiliae* AZT80; YP_005391357, YP_005391134: *Rickettsia montanensis* OSU 85-930; YP_004885050: *Rickettsia japonica* YH; YP_004764600: *Rickettsia heilongjiangensis* 054; YP_005066035: *Rickettsia slovaca* 13-B; VBIRicCon229600_1196: *Rickettsia conorii* subsp. *indica* ITTR; NP_360559: *Rickettsia conorii* Malish 7; YP_005393160: *Rickettsia parkeri* Portsmouth; YP_002845446: *Rickettsia africae* ESF-5; VBIRicSib225156_1146: *Rickettsia sibirica* subsp. *mongolitimonae* HA-91; ZP_00142926: *Rickettsia sibirica* 246; VBIRicPea48268_0721, VBIRicPea48268_0720: *Rickettsia peacockii* Rustic; YP_005301006: *Rickettsia philipii* 364D; YP_005295628: *Rickettsia rickettsii* Hlp#2; YP_001495020: *Rickettsia rickettsii* Sheila Smith.

## Supporting Information

Figure S1 **Phylogeny estimation of** ***Rickettsia*** **Pat1 and Pat1-like patatin phospholipases (cd07199).** The conserved domain cd07199 includes PNPLA8, PNPLA9, and Pat17 patatin-like phospholipases. See text for alignment and tree-building methods. Tree is final optimization likelihood: (−61591.367104) using WAG substitution model with GAMMA and proportion of invariant sites estimated. Alpha: 1.292889, invar: 0.001643, tree length: 73.651552. Branch support is from 1000 bootstrap pseudoreplications.
(PDF)

Figure S2 **Phylogeny estimation of** ***Rickettsia*** **Pat2 and Pat2-like patatin phospholipases (cd07207).** The conserved domain cd07207 is typified by the secreted bacterial proteins ExoU (*Pseudomonas aeruginosa*) and VipD (*Legionella pneumophila*). See text for alignment and tree-building methods. Tree is final optimization likelihood: (−68619.030488) using WAG substitution model with GAMMA and proportion of invariant sites estimated. Alpha: 1.467663, invar: 0.001235, tree length: 85.978288. Branch support is from 1000 bootstrap pseudoreplications.
(PDF)

Figure S3 **Purified recombinant proteins for phospholipase A2 assay.** Imperial Protein Stained (Pierce) 4 to 12% Tris-glycine precast gel (Invitrogen) using 1×Tris-glycine-SDS running buffer (BioRad). Purified recombinant proteins (including C-terminal *myc* epitope and 6×His tag) expressed in *E. coli* TOP10 cells shown: **Lane 1**, Pat2 (70.2 kD from pTrc-522HS); **Lane 2**, Pat1 (60.1 kD from pTrc-590HS); **Lane 3**, Pat1-SD (60.1 kD from pTrc-590SD); **Lane M**, SeeBlue Plus2 prestained protein standard (Invitrogen). The apparent mass of Pat1 and Pat1-SD on SDS-PAGE gel is slightly higher than their predicted molecular mass (60.1 kD), possibly due to the acidic nature of this protein (pI 4.9), as acidic protein migrates with altered masses [55].
(PDF)

Table S1 **NCBI and PATRIC accession numbers for** ***Rickettsia*** **Pat1 and Pat2 sequences.**
(PDF)
