# Supplementary figures and images for: Rickettsia typhi Possesses Phospholipase A2 Enzymes that Are Involved in Infection of Host Cells
Source: PLoS Pathog. 2013 Jun 20;9(6):e1003399. doi: 10.1371/journal.ppat.1003399 (PMC3688537; doi:10.1371/journal.ppat.1003399)

Figure S1

Pat1

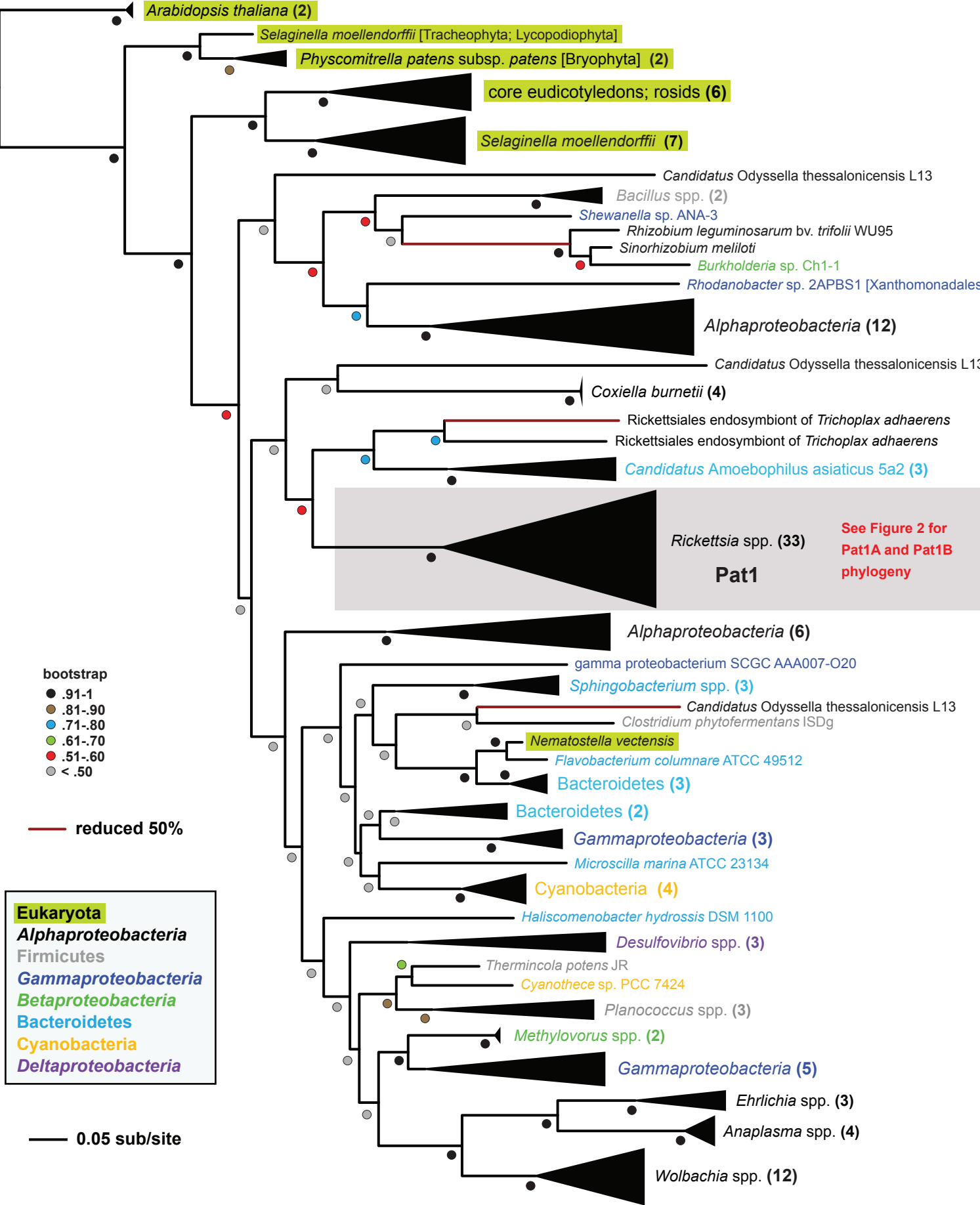

Supplement: Figure S1 — Phylogeny estimation of Rickettsia Pat1 and Pat1-like patatin phospholipases (cd07199). The conserved domain cd07199 includes PNPLA8, PNPLA9, and Pat17 patatin-like phospholipases. See text for alignment and tree-building methods. Tree is final optimization likelihood: (−61591.367104) using WAG substitution model with GAMMA and proportion of invariant sites estimated. Alpha: 1.292889, invar: 0.001643, tree length: 73.651552. Branch support is from 1000 bootstrap pseudoreplications. (PDF) [file ppat.1003399.s001.pdf]

Figure S2

Pat2

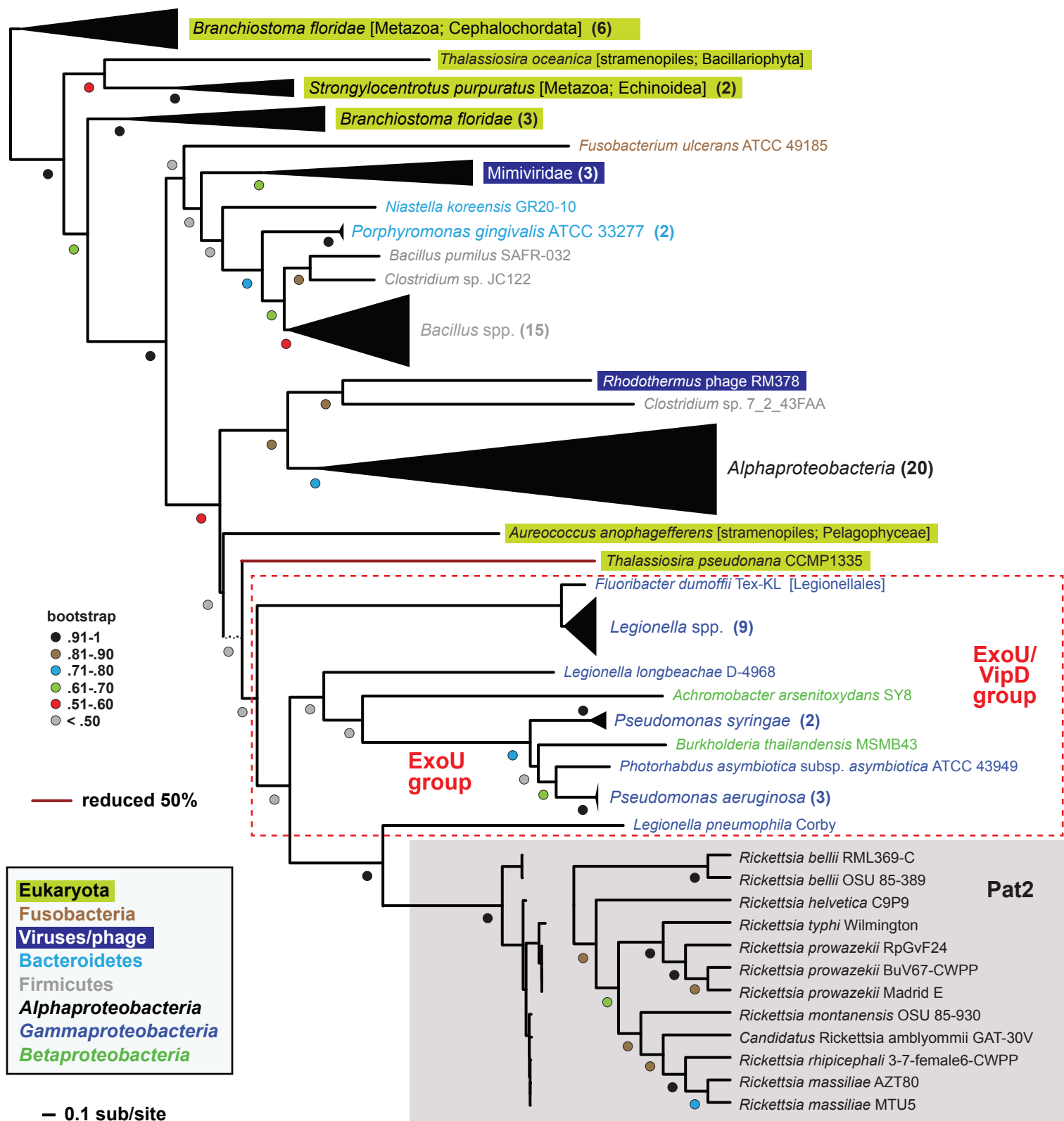

Supplement: Figure S2 — Phylogeny estimation of Rickettsia Pat2 and Pat2-like patatin phospholipases (cd07207). The conserved domain cd07207 is typified by the secreted bacterial proteins ExoU (Pseudomonas aeruginosa) and VipD (Legionella pneumophila). See text for alignment and tree-building methods. Tree is final optimization likelihood: (−68619.030488) using WAG substitution model with GAMMA and proportion of invariant sites estimated. Alpha: 1.467663, invar: 0.001235, tree length: 85.978288. Branch support is from 1000 bootstrap pseudoreplications. (PDF) [file ppat.1003399.s002.pdf]

Figure S3

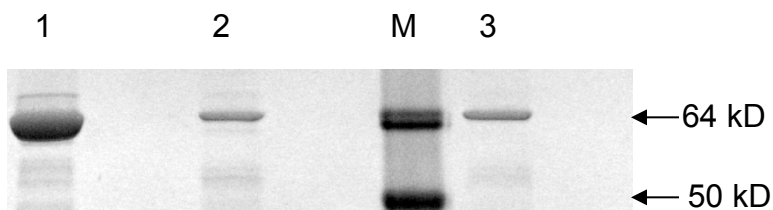

Supplement: Figure S3 — Purified recombinant proteins for phospholipase A2 assay. Imperial Protein Stained (Pierce) 4 to 12% Tris-glycine precast gel (Invitrogen) using 1×Tris-glycine-SDS running buffer (BioRad). Purified recombinant proteins (including C-terminal myc epitope and 6×His tag) expressed in E. coli TOP10 cells shown: Lane 1, Pat2 (70.2 kD from pTrc-522HS); Lane 2, Pat1 (60.1 kD from pTrc-590HS); Lane 3, Pat1-SD (60.1 kD from pTrc-590SD); Lane M, SeeBlue Plus2 prestained protein standard (Invitrogen). The apparent mass of Pat1 and Pat1-SD on SDS-PAGE gel is slightly higher than their predicted molecular mass (60.1 kD), possibly due to the acidic nature of this protein (pI 4.9), as acidic protein migrates with altered masses [55]. (PDF) [file ppat.1003399.s003.pdf]
