# Supplementary material for: Rickettsia typhi Possesses Phospholipase A2 Enzymes that Are Involved in Infection of Host Cells
Source: PLoS Pathog. 2013 Jun 20;9(6):e1003399. doi: 10.1371/journal.ppat.1003399 (PMC3688537; doi:10.1371/journal.ppat.1003399)
Supplement: Table S1 — NCBI and PATRIC accession numbers for Rickettsia Pat1 and Pat2 sequences. (PDF) [file ppat.1003399.s004.pdf]

Table S1. NCBI and PATRIC accession numbers for *Rickettsia* Pat1 and Pat2 sequences.

Pat1 sequences

Taxon	NCBI	PATRIC
Rickettsia bellii RML369-C	YP_538013<br>YP_538012	VBIRicBel102610_0959<br>VBIRicBel102610_0958
Rickettsia bellii OSU 85-389	YP_001495950	VBIRicBel35792_0564
Rickettsia canadensis McKiel	YP_001492093	VBIRicCan89738_0393
Rickettsia canadensis CA410	YP_005299400	VBIRicCan238964_0359
Rickettsia helvetica C9P9		VBIRicHel217856_0676
Rickettsia felis URRWXCal2<br>pRF	YP_246376<br>YP_247427	VBIRicFel64634_0421<br>VBIRicFel64634_1695
Rickettsia australis Cutlack	YP_005414840	VBIRicAus231019_0707
Rickettsia akari Hartford	YP_001493712	VBIRicAka50705_0974
Rickettsia typhi Wilmington	YP_067537	VBIRicTyp34752_0611
Rickettsia prowazekii GvV257	YP_005405102	VBIRicPro231244_0891
Rickettsia prowazekii Madrid E	NP_220970	VBIRicPro72556_0620
Rickettsia endosymbiont of *Ixodes scapularis*	ZP_04699958	VBIRicEnd40569_1815
Candidatus Rickettsia amblyommii GAT-30V<br>pMCE_1	YP_005365687<br>YP_005353138	
Rickettsia rhipicephali 3-7-female6-CWPP	YP_005390723	VBIRicRhi233851_1199
Rickettsia massiliae MTU5	YP_001499568	VBIRicMas83254_1188
Rickettsia massiliae AZT80	YP_005301963	VBIRicMas238520_0724
Rickettsia montanensis OSU 85-930	YP_005391357	VBIRicMon232555_0371
Rickettsia japonica YH	YP_004885050	VBIRicJap83739_1100
Rickettsia heilongjiangensis 054	YP_004764600	VBIRicHei193551_1060
Rickettsia slovaca 13-B	YP_005066035	VBIRicSlo180092_1103
Rickettsia conorii subsp. *indica* ITTR		VBIRicCon229600_1196
Rickettsia conorii Malish 7	NP_360559	VBIRicCon45613_1049
Rickettsia parkeri Portsmouth	YP_005393160	VBIRicPar233447_1088
Rickettsia africae ESF-5	YP_002845446	VBIRicAfr6986_1031
Rickettsia sibirica subsp. *mongolitimonae* HA-91		VBIRicSib225156_1146

Taxon	NCBI	PATRIC
Rickettsia sibirica 246	ZP_00142926	VBIRicSib27963_1309
Rickettsia peacockii Rustic	YP_002916515	VBIRicPea48268_0721<br>VBIRicPea48268_0720
Rickettsia philipii 364D	YP_005301006	VBIRicPhi124131_1077
Rickettsia rickettsii Hlp#2	YP_005295628	VBIRicRic236174_1074
Rickettsia rickettsii Sheila Smith	YP_001495020	VBIRicRic5337_1062

Pat2 sequences

Taxon	NCBI	PATRIC
Rickettsia bellii RML369-C	YP_538517	VBIRicBel102610_1347
Rickettsia bellii OSU 85-389	YP_001495691	VBIRicBel35792_0274
Rickettsia helvetica C9P9		VBIRicHel217856_0986
Rickettsia typhi Wilmington	YP_067473	VBIRicTyp34752_0547
Rickettsia prowazekii GvV257	YP_005413959	VBIRicPro237744_0530
Rickettsia prowazekii Madrid E	NP_220907	VBIRicPro72556_0544
Candidatus Rickettsia amblyommii GAT-30V	YP_005365402	
Rickettsia rhipicephali 3-7-female6-CWPP	YP_005390510	VBIRicRhi233851_0919
Rickettsia massiliae MTU5	YP_001499395	VBIRicMas83254_0848
Rickettsia massiliae AZT80	YP_005302159	VBIRicMas238520_0975
Rickettsia montanensis OSU 85-930	YP_005391134	VBIRicMon232555_0111
